# Pigeonpea—*Phytophthora cajani* isolates characterization and assessment of resistance in elite pigeonpea germplasm and breeding lines

**DOI:** 10.3389/fmicb.2026.1910854

**Published:** 2026-08-12

**Authors:** B. Nandhini, Shamarao Jahagirdar, Ramanagouda Gaviyappanavar, Mamta Sharma

**Affiliations:** 1 Department of Plant Pathology, University of Agricultural Sciences, Dharwad, Karnataka, India; 2 Legumes Pathology, Accelerated Crop Improvement, International Crops Research Institute for the Semi-Arid Tropics, Patancheru, Telangana, India; 3 Keladi Shivappa Nayaka University of Agricultural and Horticultural Sciences, Shimoga, Karnataka, India

**Keywords:** oomycetes, Phytophthora blight, pigeonpea, stable resistance, variability

## Abstract

Phytophthora blight (PB), caused by *Phytophthora cajani*, is a key constraint limiting pigeonpea productivity across the tropics and subtropics. Frequent PB outbreaks under erratic rainfall and warm temperatures intensifies the need for a holistic understanding of pathogen variability and stable host resistance. This study evaluated the morpho-cultural, physiological, and molecular diversity of *P. cajani* and investigated pigeonpea genotypes for resistance against representative isolates. Twenty-two isolates collected from diverse agro-ecological zones of India were purified, and six representative isolates (Pc-04, Pc-06, Pc-07, Pc-08, Pc-16 and Pc-20) were selected for detailed characterization. Significant morpho-cultural variability was observed across nine solid media, with TEA, V8J and OMA supporting maximum mycelial growth. Colony morphology, sporangial papillation, size and abundance varied among isolates. Physiologically, isolates differed markedly in pH and temperature responses, with optimum growth recorded between pH 5.5–8.0 and at 25–30 °C. Growth declined sharply below pH 4.5 and above pH 9.0, and no growth occurred at extreme temperatures (5–10 °C and 40 °C), although viability was retained upon shifting from cooler to permissive conditions. ITS-based phylogenetic analysis confirmed all isolates as *P. cajani* with high sequence similarity and discernible phylogenetic clustering with other *Phytophthora* spp. were also established. Fourteen pigeonpea genotypes found promising in previous studies, were screened against the six isolates under greenhouse conditions. Distinct differential reactions were recorded, with ICPL 99008 and ICPL 99009 showing consistent broad-spectrum resistance to all the isolates of *P. cajani*. Isolates Pc-04 and Pc-07 exhibited high aggressiveness, whereas Pc-16 and Pc-20 were comparatively less aggressive. This integrated analysis elucidates substantial diversity within *P. cajani* populations and identifies promising pigeonpea genotypes with stable resistance.

## 1 Introduction

Pigeonpea (*Cajanus cajan* (L.) Millspaugh) is a multi-purpose and resilient legume widely cultivated across the semi-arid and sub-tropical regions worldwide. The crop is now grown in more than 50 countries spanning Asia, Africa, America and Australia (FAO, 2024). In the semi-arid tropics, pigeonpea is one of the most significant pulse crops, serving as a vital component of both food and livelihood security for millions of smallholder farmers. As a rich source of dietary protein, it makes a significant contribution to both human and livestock nutrition (Saxena et al., 2002; Musokwa and Mafongoya, 2021). Over the past decade, global pigeonpea production has averaged approximately 4.9 million tonnes annually from nearly 5.7 million hectares of cultivated area. India leads global pigeonpea production, with an impressive output accounting for approximately 77% of the cultivated area and 73% of the global grain output (FAO, 2024). Other notable global producers include Myanmar, Malawi, Tanzania, Kenya, Uganda, Nepal and Dominican Republic (FAO, 2024). This wide geographical distribution underscores pigeonpea’s adaptability and its significance in ensuring nutritional security under rainfed agricultural systems. The productivity of pigeonpea has been severely constrained by major diseases such as Fusarium wilt (FW), Sterility Mosaic Disease (SMD) and Phytophthora blight (PB) (Sharma and Ghosh, 2018). Among these, PB caused by *Phytophthora cajani,* is one of the highly detrimental emerging threats to pigeonpea production and can cause yield loss even up to 100% depending on the climatic conditions (Pande et al., 2011). The disease was first reported in India (Williams et al., 1975) and later documented in several other pigeonpea-growing regions worldwide including America, Australia, Dominican Republic, Nepal, Kenya, Panama and Puerto Rico (Pal et al., 1970; Williams et al., 1975; Kannaiyan et al., 1984; Wearing and Birch, 1988; Erwin and Riberiro, 1996; Nene and Sheila, 1996). Initially, the causal organism was identified as *P. drechsleri* f. sp. *cajani*, but subsequent taxonomic revisions recognized it as, *P. cajani* (Amin et al., 1978; Sharma et al., 2015, 2023). Considering the widespread distribution and increasing severity, PB has emerged as a major threat to pigeonpea production globally.

The increasing occurrence of PB is closely associated with variable weather patterns, particularly frequent flooding and extreme precipitation over short periods combined with warm temperatures that favors disease development regardless of cropping system, soil type, or cultivar grown (Sharma and Ghosh, 2018). The distinctive visual symptoms include, water-soaked lesions that later turn necrotic and irregular (up to 1 cm in diameter) in infected leaves. Under high humidity (>80%), brown to dark brown lesions form near the stem base, sharply contrasting with healthy tissue. These lesions expand quickly, inducing the portion of the plant above the lesion to dry out but remain attached to the plant. The severely infected stem often breaks easily at the infected site under windy conditions. Sometimes, plants develop prominent stem galls and severely affected patches become visible across the field (Pande et al., 2011; Sharma and Ghosh, 2016, 2018).

The challenge of PB management is compounded by the pathogen’s variability, complex survival structures and ability to adapt to diverse agro-ecological niches. Conventional approaches such as cultural practices and fungicides offer limited and inconsistent protection. Host resistance is widely regarded as the most sustainable management option (Balaji et al., 2025); however, its durability is compromised by the high degree of pathogen variability. Previous studies have reported differences among *P. cajani* isolates in terms of growth rate, colony morphology, pathogenicity and molecular profiles (Singh and Dubey, 2005a,b; Sharma et al., 2015; Jadesha et al., 2022). Yet, comprehensive investigations that combine cultural, physiological, and molecular characterization with host-pathogen interaction studies are scarce. Without such knowledge, resistance breeding remains largely empirical and vulnerable to breakdown.

To address this gap, the present study evaluated a diverse set of *P. cajani* isolates collected from different pigeonpea-growing regions of India. The objectives were to (i) characterize *P. cajani* isolates based on cultural, morphological, physiological and molecular traits, and (ii) evaluate pigeonpea genotypes for resistance against representative isolates to identify stable resistant sources. By integrating phenotypic and molecular variability with host responses, this work provides insights into the pathogen’s diversity and offers a foundation for developing effective and durable resistance strategies in pigeonpea improvement programs.

## 2 Materials and methods

The study was conducted in the Legume Pathology laboratory and greenhouse, ICRISAT, Patancheru, India. Samples for the study were obtained during the survey for the disease status from various pigeonpea growing areas of India, spanning over the years from 2012 to 2024.

### 2.1 Collection and isolation of *Phytophthora cajani*

A total of twenty-two symptomatic pigeonpea stem samples were collected from major pigeonpea-growing regions of India (Table 1). Pigeonpea stem signifying characteristic symptoms of PB were sourced in paper bags. Sample bits were then surface sterilized using 1% sodium hypochlorite, rinsed in sterile distilled water, and plated on Vegetable-8-Juice agar (V8J) media amended with PARP antibiotics (pimarcin 400 μL; ampicillin 250 mg; rifampicin 1,000 μL; and pentachloronitrobenzene 5 mlL^−1^ media). After incubation at 25 ± 2 °C for 5–7 days, single hyphal tip cultures were established on 20% tomato extract agar (tomato extract 200 mL, CaCO_3_ 2 g and agar 20 gL^−1^) and incubated at 25 ± 2 °C. To sustain aggressiveness, the pathogen was sub-cultured at 30-day intervals and re-isolated every 2–3 months by passing the pathogen through the host (Sharma and Ghosh, 2016). Representative isolates (Pc-04, Pc-06, Pc-07, Pc-08, Pc-16 and Pc-20) that reflect different agroecological zones were selected for detailed variability studies.

**Table 1:** Details of *P. cajani* isolates collected from various pigeonpea growing areas over the years.

S. No.	Isolate Name	State	Location	Soil type	Year of isolation	NCBI accession No.
1	Pc-01	Telangana	ICRISAT	Red	2012	KJ010534
2	Pc-02	Telangana	ICRISAT	Black	2012	KJ010535
3	Pc-03	Telangana	ICRISAT	Red	2012	KJ010536
4	Pc-04*	Telangana	ICRISAT	Black	2012	KJ010537
5	Pc-05	Telangana	ICRISAT	Black	2012	KJ010538
6	Pc-06*	Telangana	Mahbubnagar	Black	2013	KJ622200
7	Pc-07*	Telangana	Adilabad	Black	2013	KJ622201
8	Pc-08*	UP	Varanasi	Sandy loam	2013	PX770773
9	Pc-09	Telangana	ICRISAT	Red	2013	KJ622202
10	Pc-10	Telangana	ICRISAT	Red	2013	KJ622203
11	Pc-11	Telangana	ICRISAT	Black	2013	KJ622204
12	Pc-12	Telangana	ICRISAT	Black	2013	KJ622205
13	Pc-13	Telangana	ICRISAT	Black	2013	KJ622206
14	Pc-14	Telangana	ICRISAT	Black	2013	KJ622207
15	Pc-15	Telangana	ICRISAT	Black	2013	KJ622208
16	Pc-16*	Punjab	Ludhiana	Sandy loam	2014	PX830351
17	Pc-17	Telangana	ICRISAT	Black	2017	PX802715
18	Pc-18	Telangana	ICRISAT	Black	2019	PX926111
19	Pc-19	Telangana	ICRISAT	Red	2023	PX928916
20	Pc-20*	Telangana	ICRISAT	Red	2023	PX929573
21	Pc-21	Telangana	ICRISAT	Black	2024	PX929576
22	Pc-22	Telangana	ICRISAT	Black	2024	PX929577

* Indicates the representative isolates used in this study.

### 2.2 Morpho-cultural variability of *Phytophthora cajani* isolates

The cultural and morphological characterization of six-representative *P. cajani* isolates were performed. Mycelial discs of 6 mm diameter of each isolate of *P. cajani* were taken from a 7 day-old actively growing colony and were assessed on nine solid media viz., Czapek Dox Agar (CDA), Corn Meal Agar (CMA), Malachite Green Agar (MGA), Oat Meal Agar (OMA), Peptone Agar (PA), Potato Dextrose Agar (PDA), Rose Bengal Agar (RBA), Tomato Extract Agar (TEA) and V8J, contained in a 90 mm petriplate. After 6 days of incubation at 25 ± 2 °C, the isolates were examined for radial growth, colony color and colony texture. Induction of sporangia in *P. cajani* isolates was performed adopting the protocol standardized by Sharma and Ghosh (2016). The morphological characteristics such as presence of hyphal swellings, production of chlamydospores, size (μm), shape and type of sporangia and the abundance of zoospores were observed using EVOS M5000 Imaging System, with samples mounted on slides at 20X magnification. All observations were recorded in triplicate for each isolate.

### 2.3 Physiological variability of *Phytophthora cajani* isolates

#### 2.3.1 Effect of pH on the mycelial growth and sporulation of *Phytophthora cajani*

To determine the effect of pH, TEA media was adjusted to 15 different pH levels (3.5 to 10.5 at 0.5 units interval) using 1N HCl or 1N NaOH. After inoculation with 7 mm discs of representative isolates, plates were incubated at 25 ± 2 °C for 6 days and radial growth was measured daily and growth rate was determined. To ascertain the influence of pH on the sporulation, pond water covering the same pH range (3.5–10.5) was used to induce sporulation in Pc-04 following the protocol of Sharma and Ghosh (2016), and zoospore production was quantified microscopically. Each treatment was carried out in triplicate.

#### 2.3.2 Temperature optima for the mycelial growth of *Phytophthora cajani*

To evaluate the thermal sensitivity of *P. cajani*, TEA plates inoculated with representative isolates were incubated under eight temperature regimes (5, 10, 15, 20, 25, 30, 35, and 40 °C) using controlled incubators. Radial growth was quantified daily for six consecutive days by measuring colony diameter. For precise determination of the cardinal temperatures, isolate Pc-04 was further incubated across narrower ranges (16–19 °C and 36–39 °C). Plates showing no visible growth at extreme temperatures (5, 10, 15, and 40 °C) were subsequently shifted to 25 °C to assess viability. All treatments were conducted in triplicate. Growth–temperature relationships were established by constructing radial growth curves against incubation temperature.

### 2.4 Molecular characterization

#### 2.4.1 DNA extraction and sequencing

The internal transcribed spacer (ITS1 and ITS2) regions, including the 5.8S rDNA of each isolate, were sequenced for molecular identification. Mycelial plugs (~6 mm) from TEA cultures were transferred into 25 mL of 20% tomato juice broth in 100 mL flasks and incubated for 3–4 days at 25 °C. Mycelial mats were harvested, blotted dry on sterile filter paper, and stored at −80 °C. Genomic DNA was isolated using the CTAB method (Doyle and Doyle, 1990). DNA integrity was checked on 0.8% (w/v) agarose gels stained with SYBR Safe (0.2 μg/mL) and visualized under UV illumination. DNA concentration and purity were quantified spectrophotometrically (NanoDrop) using OD260/280 ratios, and samples were stored at −20 °C until use. PCR amplification of the ITS region was performed using primers ITS1 (forward) and ITS4 (reverse) in 50 μL reaction mixtures containing 5 μL 10 × PCR buffer, 3.0 μL MgCl_2_ (50 mM), 1.0 μL dNTP mix (10 mM; 2.5 mM each), 0.5 μL of each primer (10 mM), 0.5 μL Taq DNA polymerase (5 U/μL; Invitrogen, United States), 1.0 μL template DNA, and nuclease-free water. Amplification was carried out with the following thermal profile: 94 °C for 5 min; 35 cycles of 94 °C for 30 s, 55 °C for 30 s and 72 °C for 1 min; followed by a final extension at 72 °C for 10 min (Sharma et al., 2015). Amplicons were visualized on 1% agarose gels and sequenced bidirectionally with ITS1 and ITS4 primers by a commercial sequencing service.

#### 2.4.2 Phylogenetic analysis

DNA sequences of all *P. cajani* isolates were aligned and consensus sequences were generated using BioEdit v.7.7.1. Each sequence was subjected to BLAST searches against the GenBank (NCBI, Bethesda, MD) and the sequences were eventually deposited in the NCBI repository. Additionally, sequences of 43 other *Phytophthora* spp. belonging to different clades (Abad et al., 2023) were retrieved from NCBI and all the sequences were aligned using ClustalW in MEGA v.12.0. Phylogenetic relationships among 22 isolates of *P. cajani* and 43 other *Phytophthora* spp. were reconstructed using MEGA v.12.0. The sequence of *Pythium aphanidermatum* retrieved from NCBI, was also included in the analysis as an outgroup. Maximum Likelihood analysis was performed under the TN93 model with gamma distribution (+G) and a proportion of invariant sites (+I). Node significance was evaluated with 1,000 bootstrap repetitions. The Newick file obtained from MEGA v.12.0. was then deposited in interactive tree of life (ITOL v.7.4.2.) for better visualization of phylogeny.

### 2.5 Screening of pigeonpea genotypes against six representative isolates of *Phytophthora cajani*

Fourteen pigeonpea genotypes, including the susceptible check ICP 7119, were evaluated against six representative isolates of *P. cajani*. The selection included entries previously reported (Sharma et al., 2023), for their contrasting reactions to *P. cajani*, along with additional breeding lines representing broader genetic variation (Table 2). Seeds were surface sterilized with 2% sodium hypochlorite for 2 min, rinsed thoroughly in sterile distilled water, and sown in plastic trays (35 × 25 × 8 cm) containing a sterilized river sand–vermiculite mixture (10:1, v/v). Each tray contained ten rows, with each row representing a genotype and consisting of eight seeds. Seedlings were raised in a greenhouse maintained at 25–28 °C under natural light for 7 days.

**Table 2:** Description of the pigeonpea genotypes used in the study.

S. No.	Genotypes	Details	Reference
1	ICP 2376	Germplasm, resistant to SMD, but highly susceptible to FW	Internal circulations (Sharma et al., 2016)
2	ICP 9174	Germplasm, resistant to all variants of FW	
3	ICPL 2011228	Breeding lines, moderately resistant-resistant to PB	
4	ICPL 20114		
5	ICPL 20124		
6	ICPL 20135		
7	ICPL 99004		
8	ICPL 99008		
9	ICPL 99009		
10	ICPL 99010		
11	ICPL 99048		
12	ICPL 99099		
13	ICPL 99102		
14	ICP 7119 (Check)	Germplasm, susceptible to PB	

For inoculum preparation, *P. cajani* was mass-cultured in 100 mL flasks containing 25 mL of 20% tomato extract broth, incubated at 25 °C in darkness for 72 hours (h). The broth was decanted and replaced with sterile pond water, and cultures were incubated in darkness for 4 h. This step was repeated, followed by a 20 h incubation. Finally, zoospores were checked under microscope and adjusted the zoospores concentration (1.5 × 10^5^ /mL^−1^) with sterilized deionized water in a hemocytometer (Sharma et al., 2015). Seven-day-old seedlings were saturated with sterile water and inoculated with the approximately 2 mL of standardized zoospore suspension per plant. Trays were maintained in the greenhouse at 28 ± 2 °C under ambient light conditions (Sharma and Ghosh, 2016). Control seedlings received sterile water only. All the plants were flooded with sterile water 3–4 times daily for 48 hours after inoculation (hai) to facilitate infection, and saturation was maintained until the completion of experiment, i.e., 120 hai Disease development was monitored daily, total number of infected plants and healthy plants were recorded. Disease severity in terms of per cent disease incidence (PDI) was recorded at four different time points (48, 72, 96, and 120 hai) by following the below formula (Pande et al., 2012).

$$\mathrm{PDI}=\frac{\text{Number of infected plants in tray}\;}{\text{Total number of plants in tray}}\times 100$$

Genotypes were categorized based on PDI as: Resistant (R, 0–10.0%), Moderately Resistant (MR, 10.1–30.0%), Susceptible (S, 30.1–70.0%), or Highly Susceptible (HS, >70.0%).

### 2.6 Data analysis

Actual data in percentage were arcsine transformed to make residuals normal and then back-transformed for the presentation of the results (Gomez and Gomez, 1984; Sokal and Rohlf, 2012). The transformed data was subjected to Two-way analysis of variance (ANOVA) to determine the independent and combined effect of variable and their level of significance. Significance of mean differences within treatments was tested by Duncan multiple range test at *p* < 0.05 level of probability. R language 4.5.1 was used for data processing, analysis and corresponding visualizations.

## 3 Results

### 3.1 Morpho-cultural variability of *Phytophthora cajani* isolates

**Figure 1:**
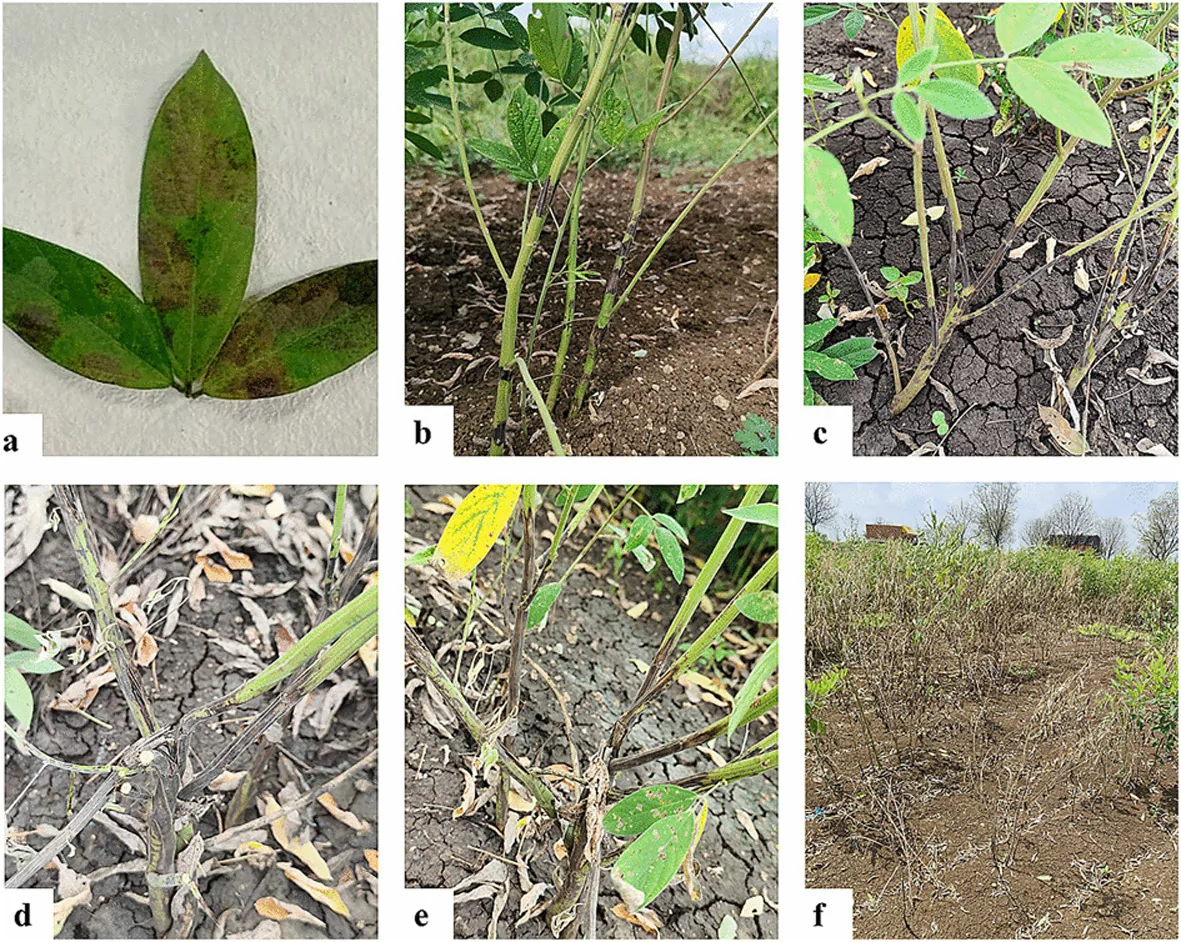
Characteristic symptoms of *Phytophthora* blight of pigeonpea. **(a)** Water soaked lesions on leaves; **(b,c)** Dark brown to black lesions on stem and petiole; **(d,e)** Broken stem at the point of infection; **(f)** Pigeonpea field mortality owing to *P. cajani* infection.

**Figure 2:**
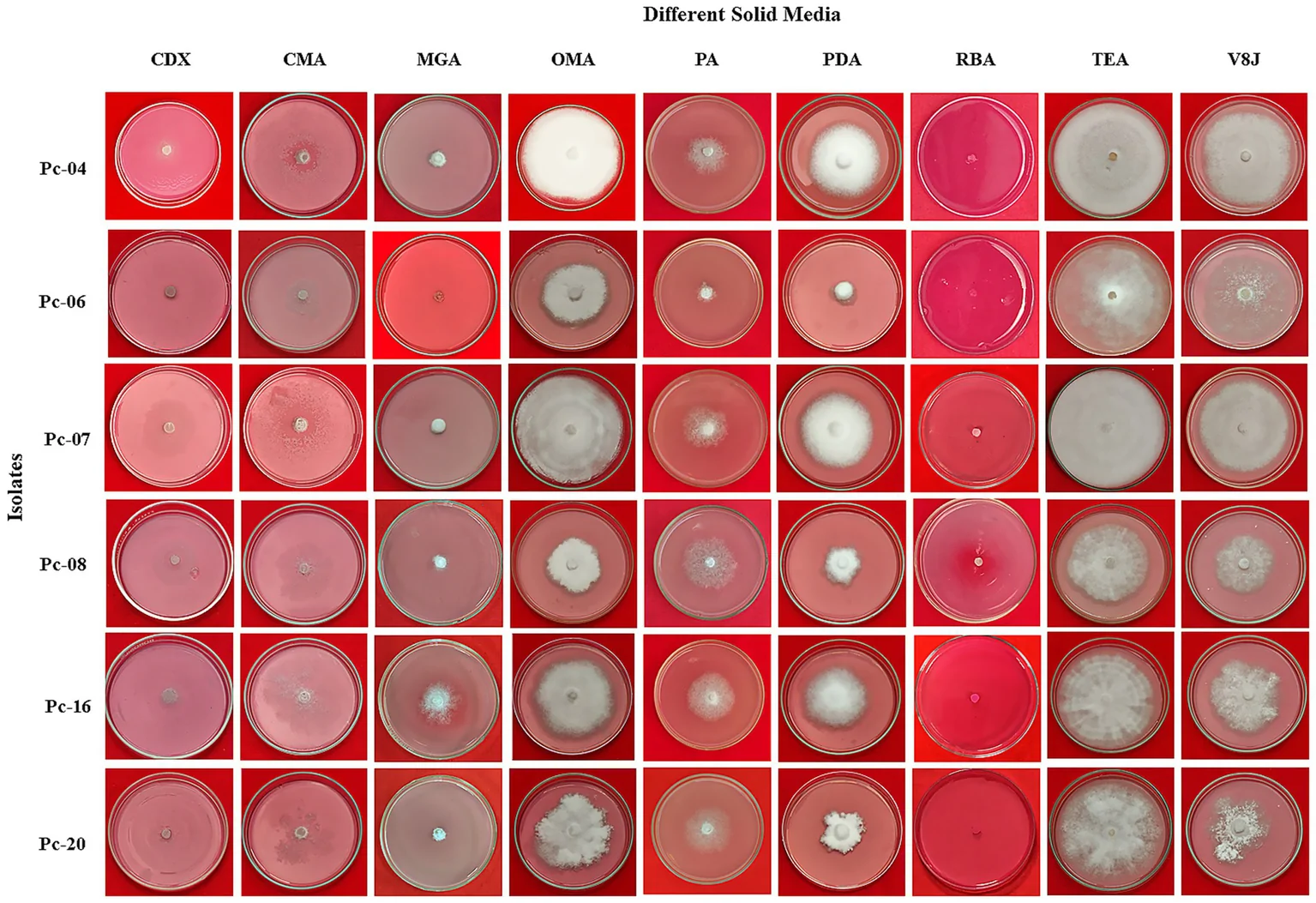
Cultural variability of six selected *P. cajani* isolates on different solid media. CDX, Czapek Dox agar; CMA, Corn meal agar; MGA, Malachite green agar; OMA, Oatmeal agar; PA, Peptone agar; PDA, Potato dextrose agar; RBA, Rose Bengal agar; TEA, Tomato extract agar; V8J, Vegetable 8 juice agar.

**Figure 3:**
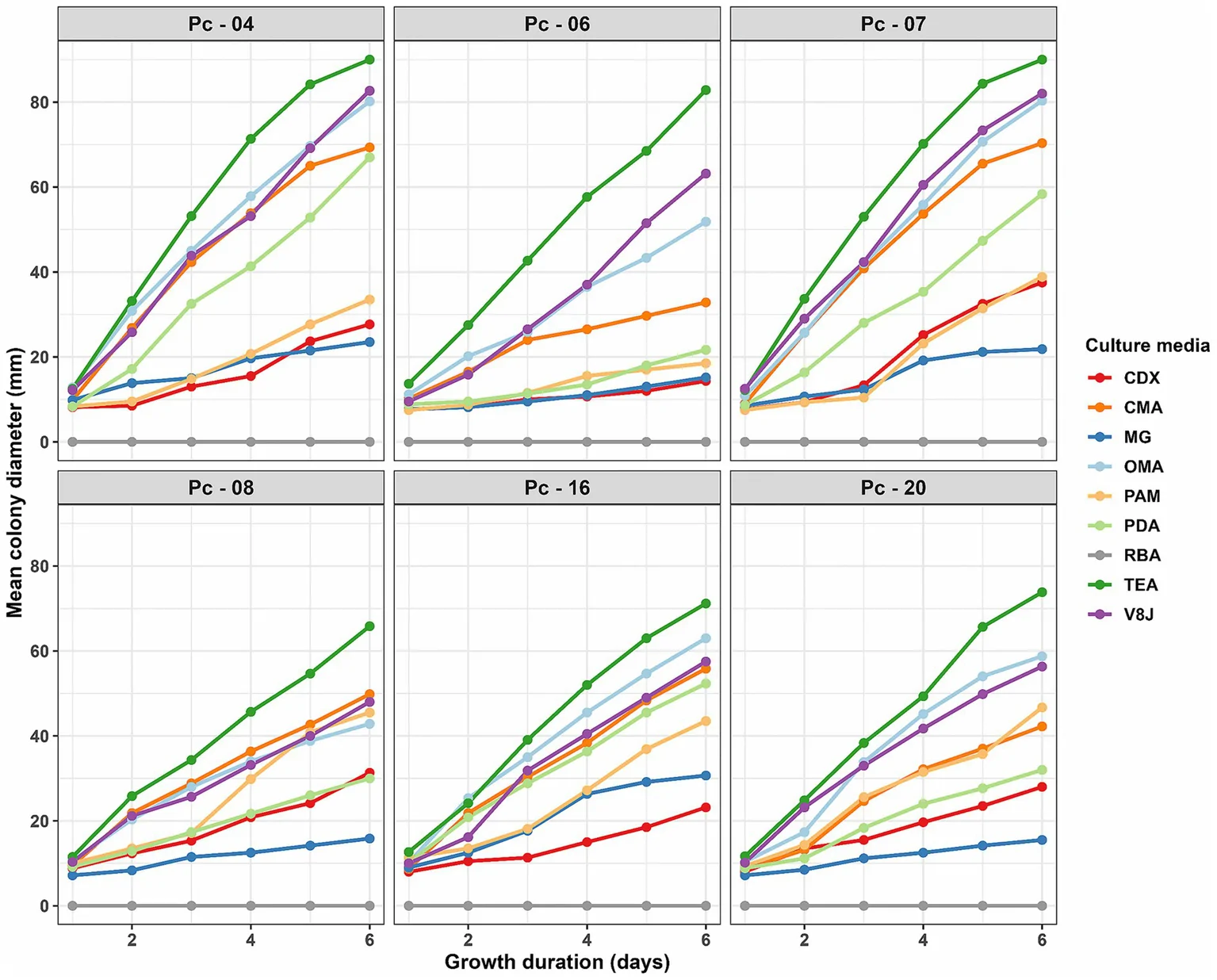
Growth dynamics of six selected *P. cajani* isolates on nine culture media over a six-day incubation period. Mean colony diameter (mm) was recorded daily and was represented in dots. Each panel represents an individual isolate and different colored lines indicate the respective culture media.

Pigeonpea plants showing typical PB symptoms (Figure 1) were collected and subjected to isolation, resulting in a total of 22 distinct isolates of *P. cajani* (Table 1). The isolates represented diverse soil types including red, black and sandy loam soils. Six representative isolates were selected based on geographical distribution, year of isolation and preliminary pathogenicity assays (Sharma, internal circulations) for detailed morpho-cultural characterization. Two-way ANOVA revealed that culture media, isolate and the media × isolate interaction significantly influenced mycelial growth (*p* < 0.01) (Table 3), indicating that the response of *P. cajani* isolates varied depending on the culture media. Although TEA generally supported the highest mycelial growth (mean of 78.94 mm), followed by V8J (64.94 mm) and OMA (62.82 mm), the magnitude of growth differed among isolates. Pc-04 and Pc-07 exhibited the most vigorous growth, attaining complete colony growth (90 mm) on TEA within 6 days after inoculation (DAI), whereas Pc-06 and Pc-20 showed relatively slower growth on most media but responded more favorably on TEA and V8J. Pc-08 and Pc-16 displayed intermediate growth across the tested media. In contrast, CDX and MG consistently supported comparatively lower mycelial growth for all isolates, while no mycelial growth was observed on RBA, confirming its unsuitability for culturing *P. cajani* (Supplementary Table 1; Figures 2, 3). Colony morphology varied widely among isolates and media. OMA, PDA and MGA consistently produced fluffy, cottony white, aerial mycelium, whereas CDX and PA supported transparent, submerged colonies. On CMA, all the isolates formed transparent colonies and the hyphae at the edge tends to be aerial. Interestingly, Pc-04 and Pc-07 formed the most luxuriant cottony white, aerial colonies on TEA, while other isolates produced dull-white, submerged colonies. On V8J, Pc-06 and Pc-08 developed dull-white submerged mycelium, whereas the remaining isolates produced cottony-white aerial colonies, indicating pronounced cultural diversity among *P. cajani* isolates (Figure 2).

**Table 3:** Analysis of variance of interaction between media and six representative *P. cajani* isolates.

Source of variation	Degrees of freedom	Sum of square	Mean sum of squares	*F* value	*p*-value
Media	7	50,709	7,244	1735.3	<2.2e-16 ***
Isolates	5	10,539	2,108	504.9	<2.2e-16 ***
Media × Isolates	35	8,941	255	61.2	<2.2e-16 ***
Residuals	96	401	4	–	–

*** Highly significant (<0.001).

**Figure 4:**
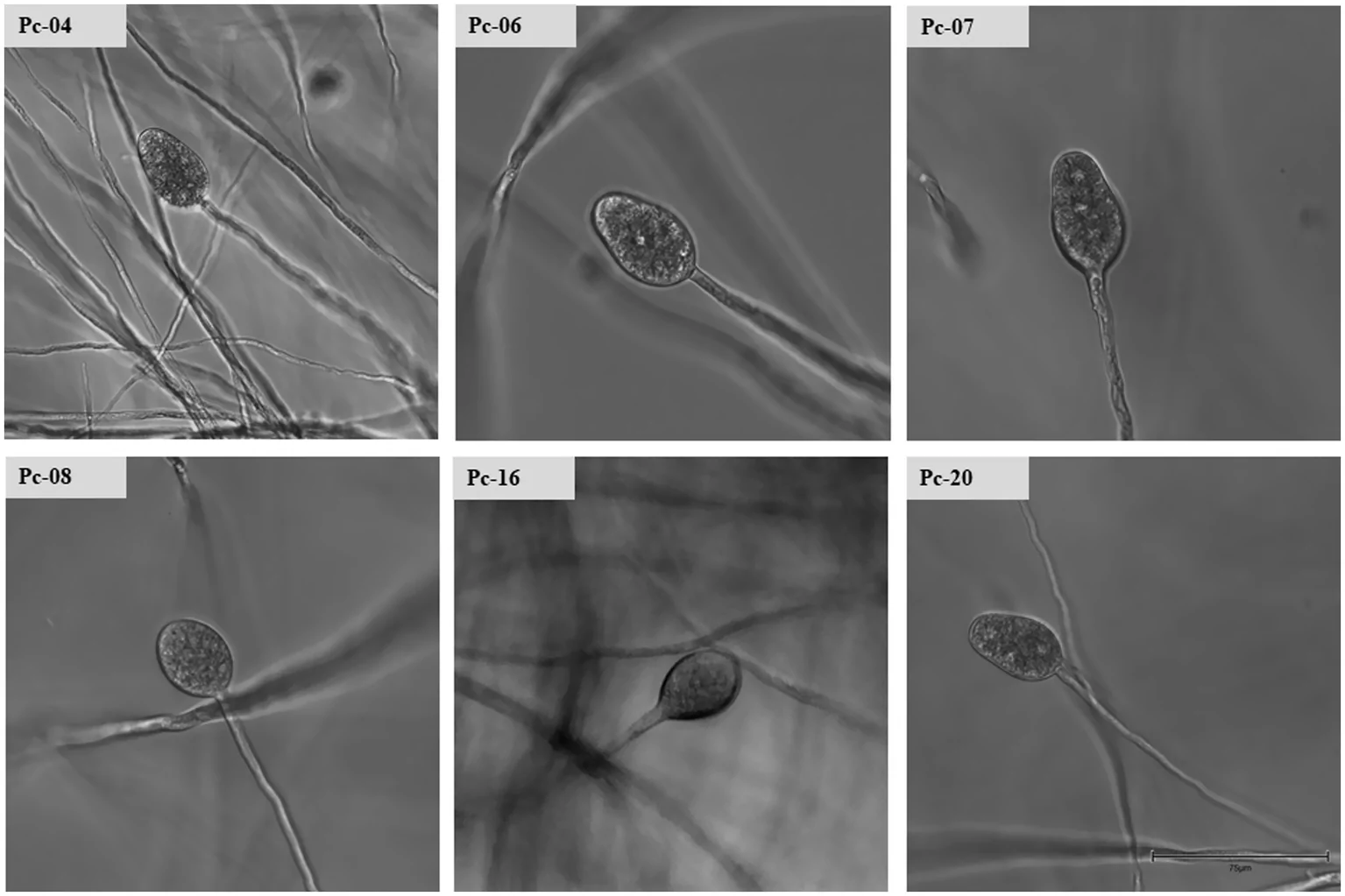
Morphological variability among six representative isolates of *P. cajani*.

All the isolates produced branched and coenocytic hyphae. Sporangia was observed to be non-papillate and non-caducous (Figure 4), but sporangial morphology showed substantial variability with shapes ranging from ovoid, obovoid, obpyriform to elongated within the *P. cajani* isolate. Sporangial lengths ranged from 35.85 μm (Pc-08) to 47.20 μm (Pc-04) and width varied between 19.40 μm (Pc-16) and 26.69 μm (Pc-04). Sporangial circumference ranged from 109.81 μm (Pc-16) to 142.44 μm (Pc-04). Among the isolates, Pc-04 recorded the largest sporangial size of 47.20 μm × 26.69 μm. Also, the isolates Pc-04 and Pc-07 produced abundant sporangia and zoospores, while Pc-06 and Pc-08 sporulated moderately. In contrast, Pc-16 and Pc-20 displayed comparatively slower sporulation rates and sparse zoospore formation. None of the isolates produced chlamydospores under the tested conditions (Supplementary Table 2).

### 3.2 Effect of pH on the mycelial growth and sporulation of *Phytophthora cajani*

**Figure 5:**
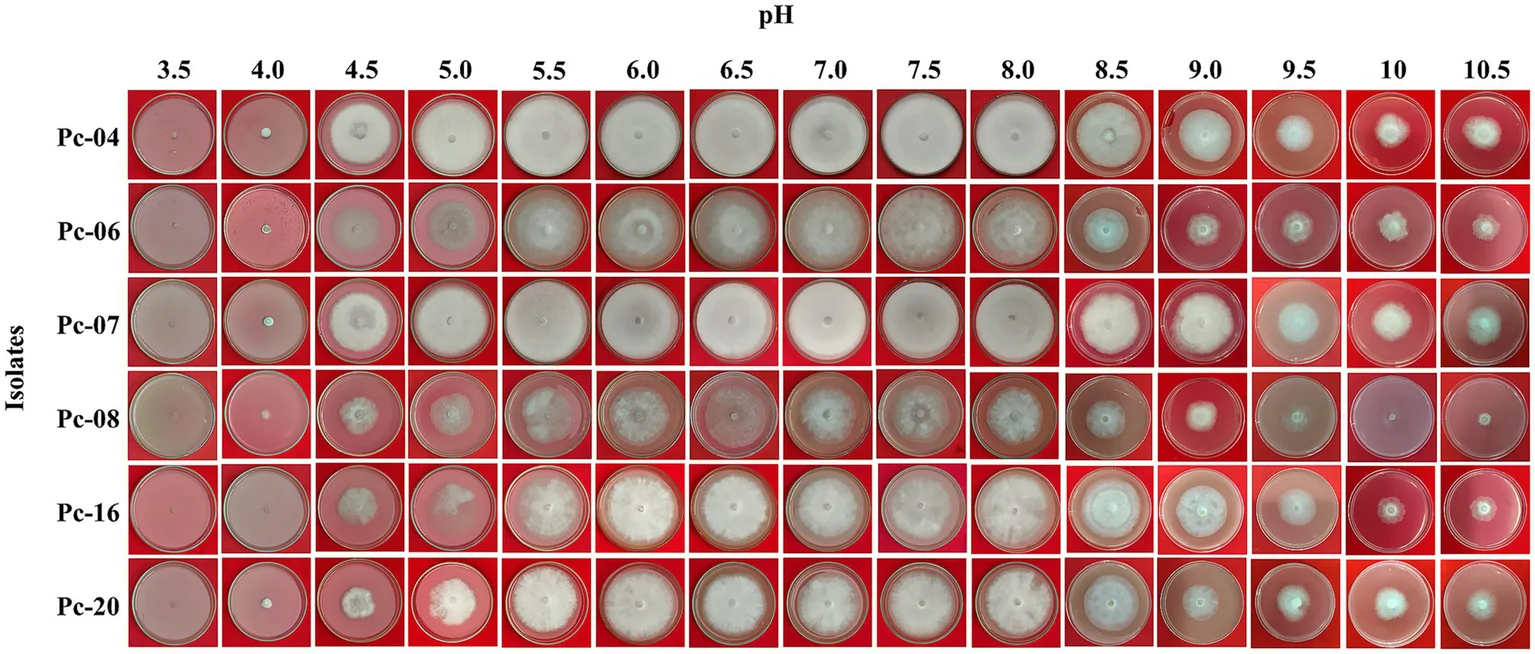
Effect of different media pH on diverse isolates of *P. cajani*.

**Figure 6:**
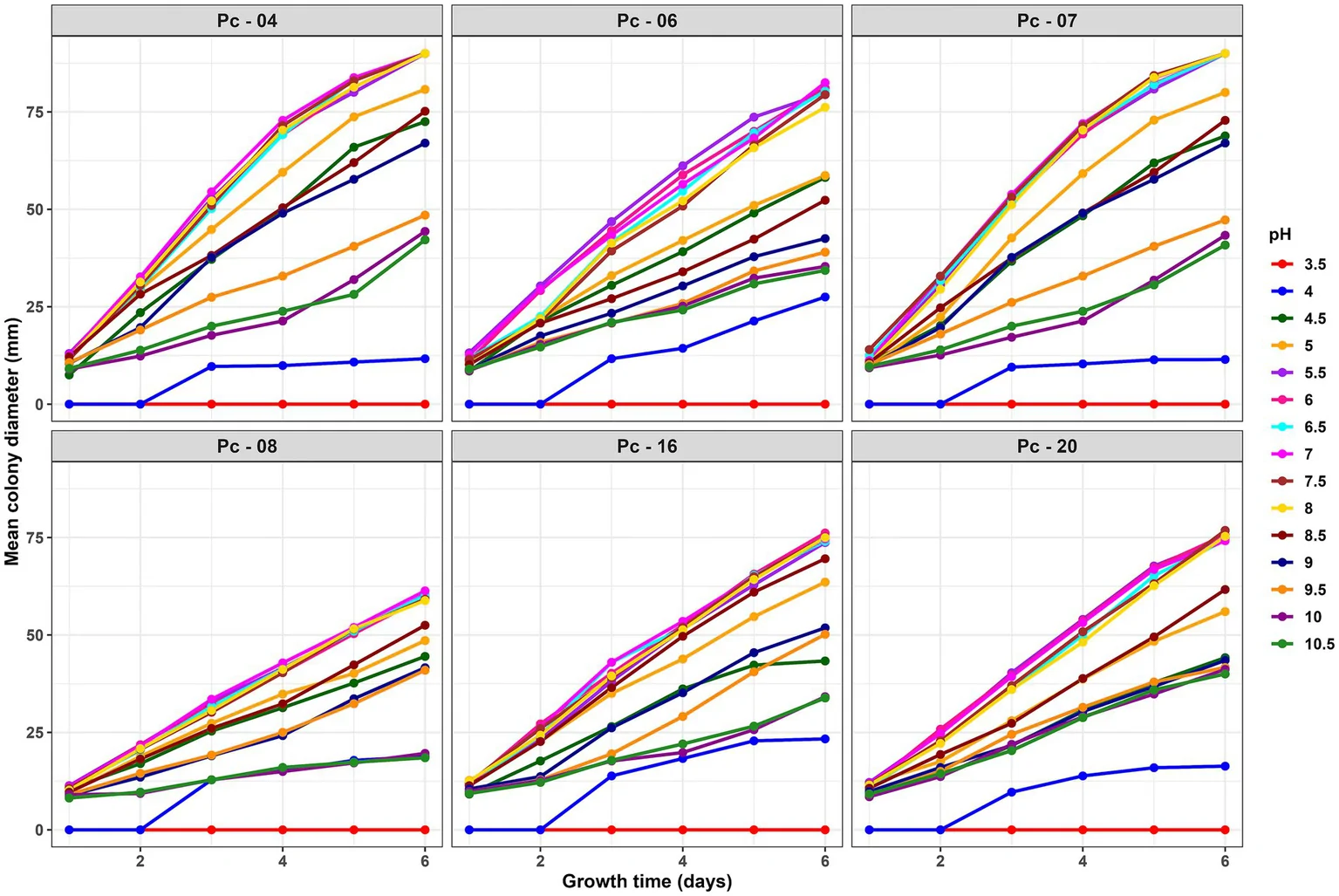
Growth curves of six *P. cajani iso*lates at different pH levels over 6 days of incubation. Points represent the mean colony diameter (mm) of replicated observations and colored lines correspond to the different pH levels tested.

Media pH had a highly significant effect (*p* < 0.01) on the mycelial growth of *P. cajani* isolates, with complete absence or very minimal growth at highly acidic condition of pH 3.5 and pH 4.0 (mean of 18.18 mm), with all isolates showing restricted radial expansion, but observed steady increase at pH 4.5 and 5.0 (55.25 mm and 64.59 mm, respectively). The highest radial growth occurred between pH 5.5 and 8.0, where means were statistically similar, indicating the optimum pH range for the pathogen (Supplementary Table 3). Growth declined gradually beyond pH 8.0 and was markedly reduced at pH ≥ 9.0 (Figures 5, 6). Table 4 reveals the significant isolate × pH interactions (*p* < 0.001), demonstrating clear physiological variability among isolates. Pc-04 and Pc-07 exhibited broad pH tolerance and achieved maximum colony diameter (90 mm) across pH 5.5–8.0, reflecting strong adaptability. Pc-06 and Pc-16 showed moderate performance, whereas Pc-08 consistently recorded the lowest growth, particularly under acidic and alkaline extremes. Sporangia and zoospores in Pc-04 were produced abundantly across the pH range of 5.0–8.5, whereas sporulation declined sharply above pH 9.0. No zoospores were observed at pH 3.5–4.0, and only scant zoospore production occurred at pH 4.5. These patterns confirm that most isolates prefer mildly acidic to near-neutral conditions for optimal development.

**Table 4:** Analysis of variance of interaction between pH and six representative *P. cajani* isolates.

Source of variation	Degrees of freedom	Sum of square	Mean sum of squares	*F* value	*p*-value
pH	13	95,517	7,347	3725.36	<2.2e-16 ***
Isolates	5	16,760	3,352	1699.57	<2.2e-16 ***
pH × Isolates	65	7,004	108	54.63	<2.2e-16 ***
Residuals	168	331	2	–	–

*** Highly significant (<0.001).

### 3.3 Temperature optima for the mycelial growth of *Phytophthora cajani*

**Figure 7:**
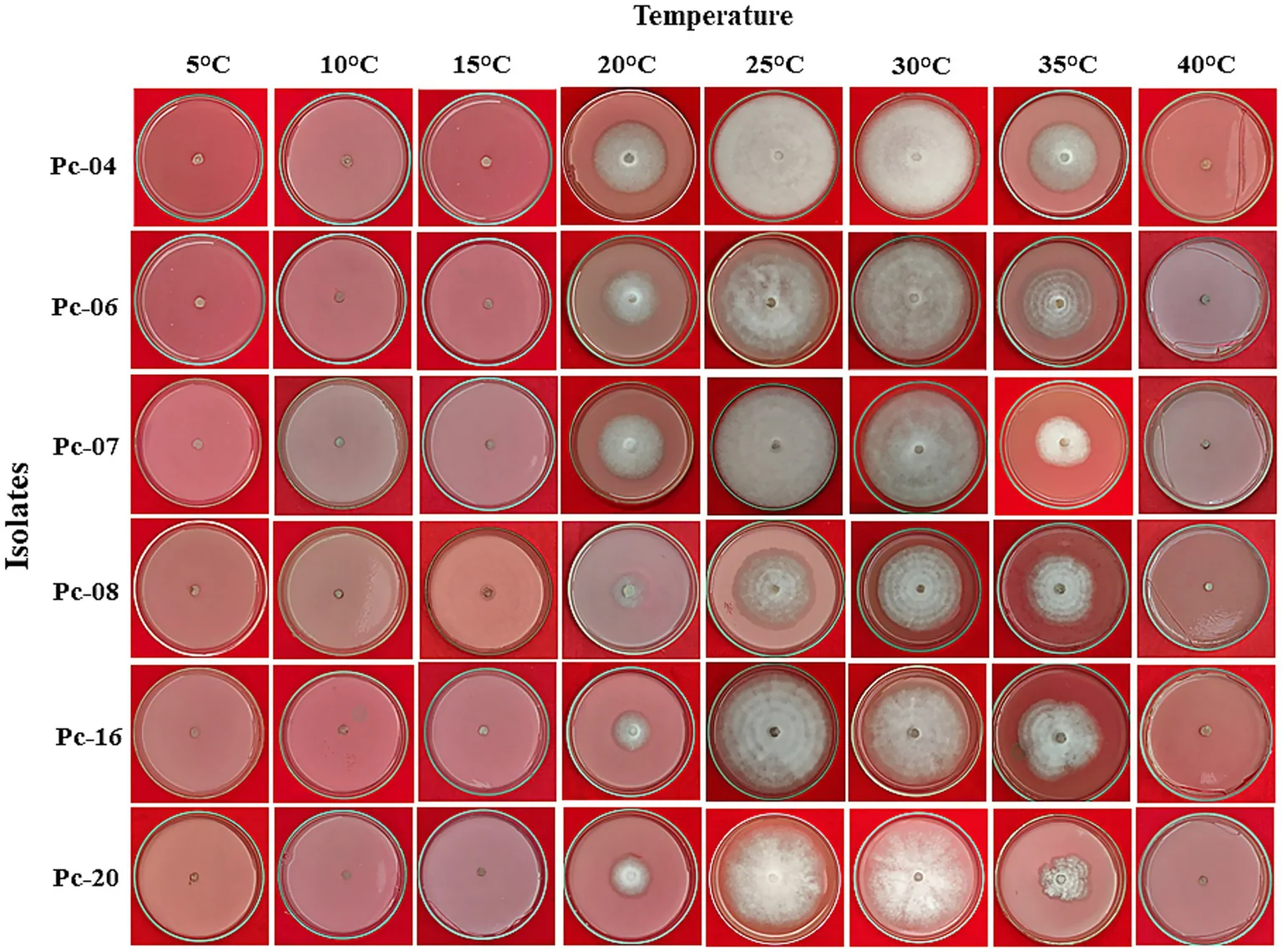
Effect of different incubation temperature on diverse isolates of *P. cajani*.

**Figure 8:**
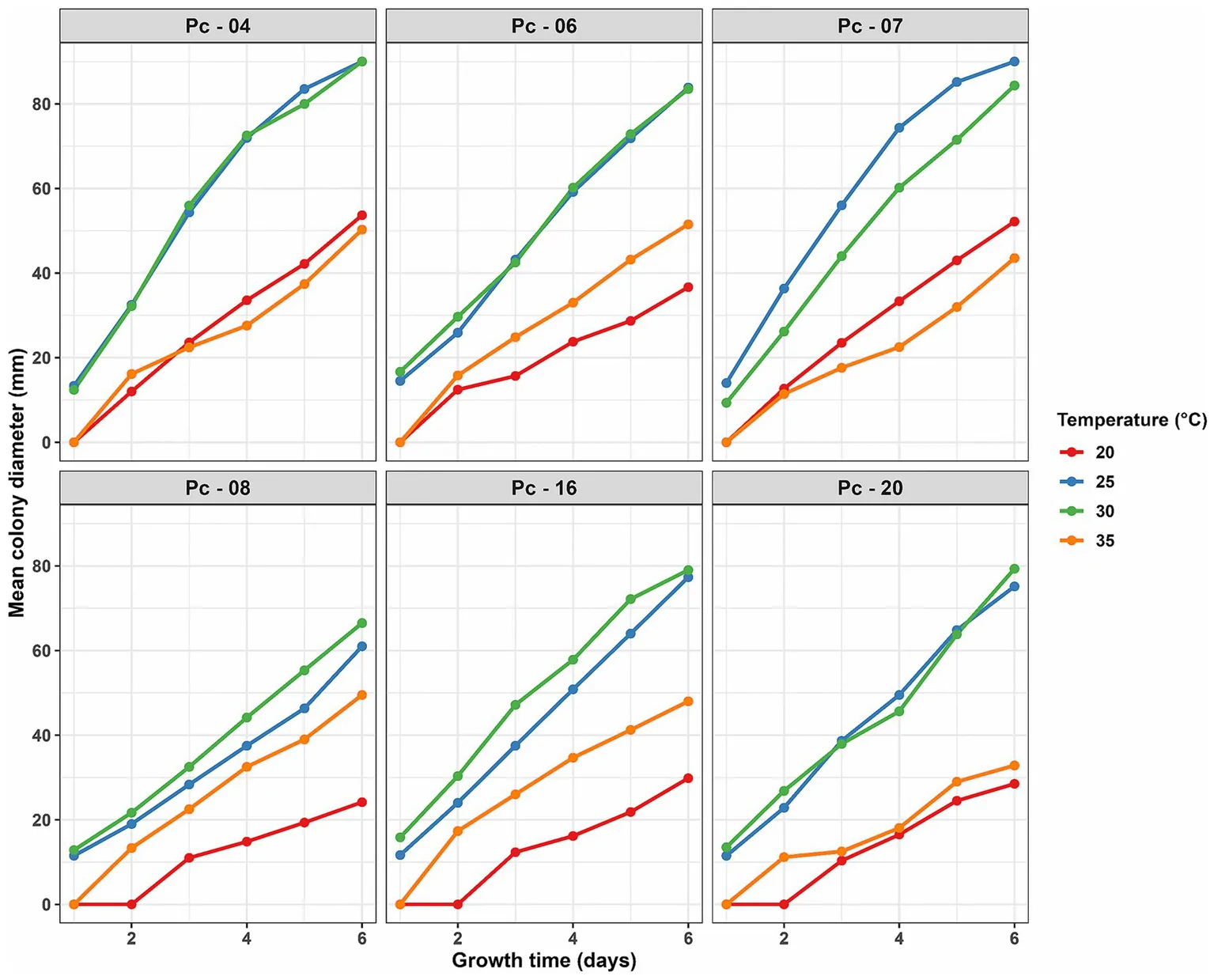
Temporal growth of six *P. cajani iso*lates at four incubation temperatures (20, 25, 30, and 35 °C). Mean colony diameter (mm) was measured over a six-day incubation period. Each panel represents one isolate, and colors denote the different incubation temperature.

Temperature exerted a significant influence (*p* < 0.01) on the mycelial development of all *P. cajani* isolates (Table 5). None of the isolates grew below 15 °C, indicating strong inhibition of mycelial activity under cold conditions. Optimal growth was consistently recorded between 25 °C and 30 °C, where the means were 79.56 mm and 80.45 mm, respectively, and were statistically at par. All isolates showed enhanced radial expansion at these temperatures, with isolates Pc-04 and Pc-07 achieving the maximum colony diameter (90 mm) at 25 °C. A sharp decline in growth occurred at 35 °C, recording the mean of 45.94 mm (Supplementary Table 4). Although most isolates continued to grow, the reduction was notable compared with the optimum temperatures of 25–30 °C (Figures 7, 8). No isolate exhibited growth at 40 °C, and cultures incubated at this temperature failed to resume growth even after being transferred to 25 °C. In contrast, the isolates incubated at 5, 10, and 15 °C regained growth capability when shifted to 25 °C.

**Table 5:** Analysis of variance of interaction between temperature and six representative *P. cajani* isolates.

Source of variation	Degrees of freedom	Sum of square	Mean sum of squares	*F* value	*p*-value
Temperature	3	27,029	9,010	1221.91	<2.2e-16 ***
Isolates	5	3,845	769	104.30	<2.2e-16 ***
Temperature × Isolates	15	2027	135	18.32	6.34e-15 ***
Residuals	48	354	7	–	–

*** Highly significant (<0.001).

**Figure 9:**
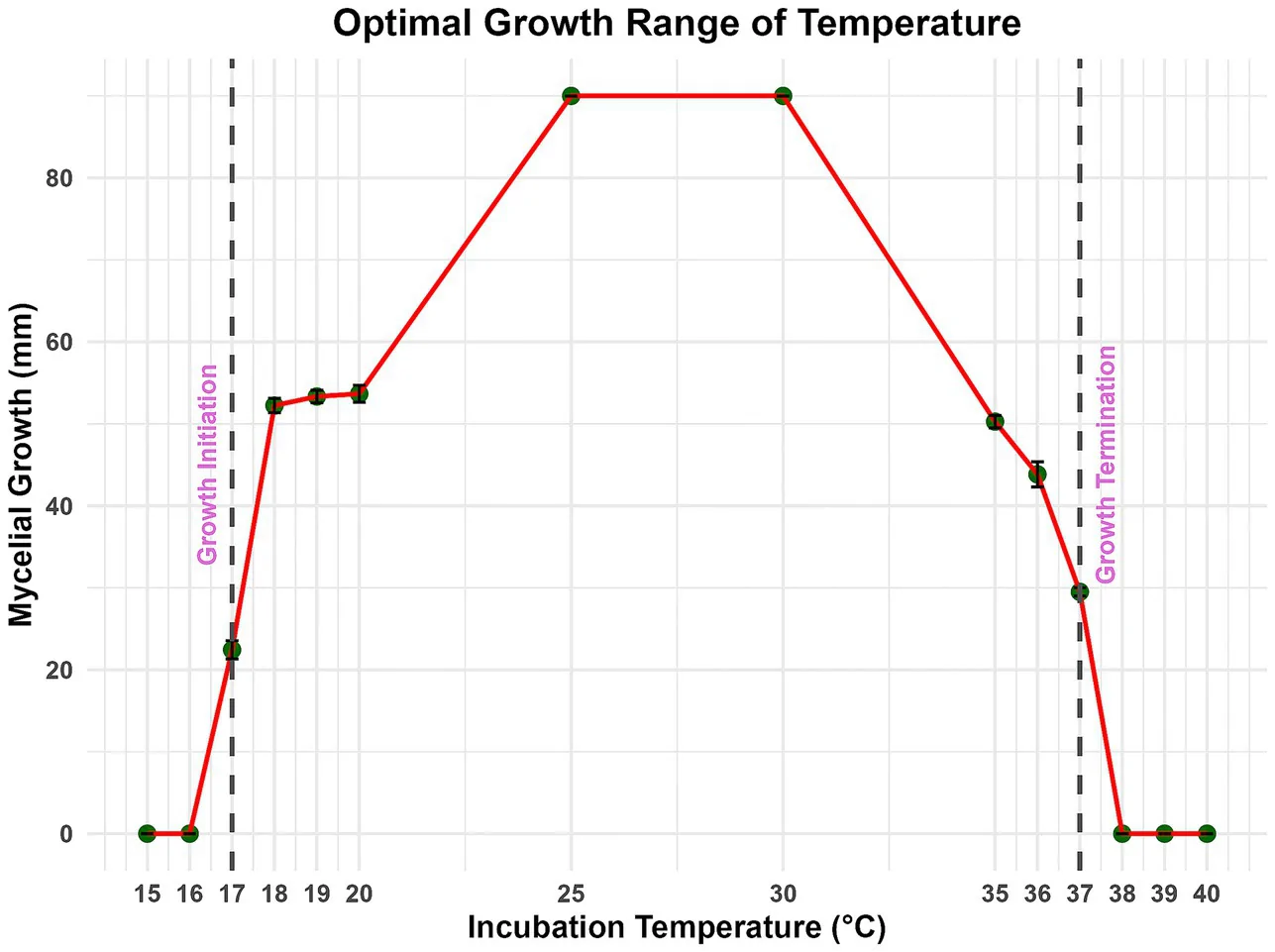
Mycelial growth curve of *P. cajani* (Pc-04) under varied incubation temperature.

To refine the cardinal temperature thresholds, isolate Pc-04 was assessed at narrower temperature intervals, and mycelial growth was recorded at 6 DAI (Figure 9). No growth occurred at 15 °C or 16 °C, confirming that the minimum temperature for initiation of mycelial growth lies above 16 °C. Mycelial growth initiated at 17 °C (22.4 mm) and increased steadily at 18–20 °C, with a similar growth response (52.2–53.7 mm). Maximum radial expansion (90 mm) was again observed at 25 °C and 30 °C, validating these as the optimum temperatures for *P. cajani*. Growth declined noticeably at 35 °C (50.3 mm) and further at 36 °C and 37 °C (43.8 mm and 29.5 mm, respectively). No growth occurred at 38–40 °C, indicating that temperatures ≥38 °C are lethal and remark the upper thermal limit for the pathogen.

### 3.4 Molecular characterization of *Phytophthora cajani* isolates

**Figure 10:**
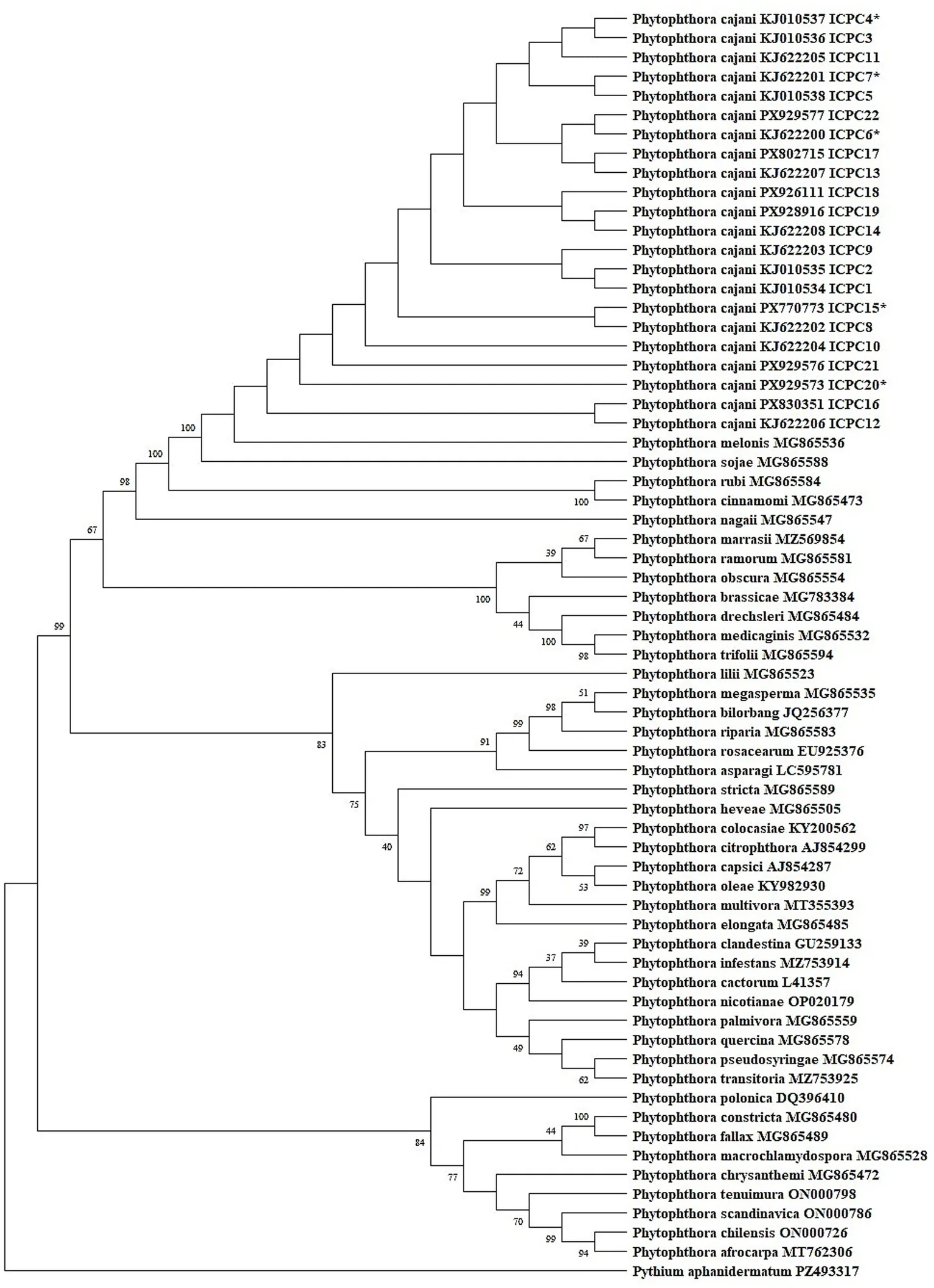
Phylogenetic relationship among the *P. cajani* isolates of this study and other *Phytophthora* spp. based on ITS sequences of 5.8S rDNA. The tree was constructed using the Maximum Likelihood method and Tamura-Nei model with gamma (G) plus invariable parameter (I). Node significance was estimated using 1,000 bootstrap replicates. Asterisks indicate the six representative isolates used in the study.

The ITS sequence of 5.8S rDNA of all the *P. cajani* isolates was amplified, sequenced and were subjected to BLASTn in NCBI. The results confirmed the sequences to be *P. cajani* and were deposited in the NCBI GenBank, obtaining accession numbers (Table 1). Pair-wise nucleotide sequence comparison among the *P. cajani* isolates revealed a high level of sequence similarity (98–100%), indicating minimal genetic variation within the species at the ITS locus. Phylogenetic analysis was performed with 22 *P. cajani* and 43 other *Phytophthora* spp. sequences retrieved from NCBI. The phylogeny showed that all the *P. cajani* sequences clustered together within a clade supported by 100% bootstrap and were most closely related to *P. melonis* and *P. sojae* (Figure 10). This clade includes the six representative isolates used for the variability analysis in this study, which were marked with asterisk. The inclusion of *Pythium aphanidermatum* and other *Phytophthora* spp. clustered in different clades clearly demonstrated the evolutionary relationship.

### 3.5 Screening of pigeonpea genotypes against six representative isolates of *Phytophthora cajani*

Analysis of variance (ANOVA) demarked highly significant effects of isolates, genotypes and their interaction (*p* < 0.001), indicating pronounced variability in isolate aggressiveness and genotype-specific resistance responses (Table 6). Disease expression varied widely across Genotype × Isolate combinations. Among the isolates, Pc-04 and Pc-07 were the most aggressive, recording PDI of 96.88 ± 5.23% and 95.31 ± 5.98%, respectively, whereas Pc-16 and Pc-20 consistently induced lower disease incidence (53.12 ± 3.61 and 62.5%, respectively) in ICP 7119 (susceptible check). These patterns clearly establish Pc-04 and Pc-07 as the most aggressive isolates in the panel (Supplementary Table 5).

**Table 6:** Analysis of variance of interaction between fourteen pigeonpea genotypes and six representative *P. cajani* isolates.

Effect	Df	Sum of square	Mean sum of squares	*F* value	*p*-value
Genotypes (G)	13	156,487	12037.5	266.867	<2.2e-16 ***
Isolates (I)	5	21,690	4338.0	96.173	<2.2e-16 ***
G × I	65	22,648	348.4	7.725	<2.2e-16 ***
Residuals	162	7,307	45.1		

Significant code: Highly significant (<0.001) “***”.

**Figure 11:**
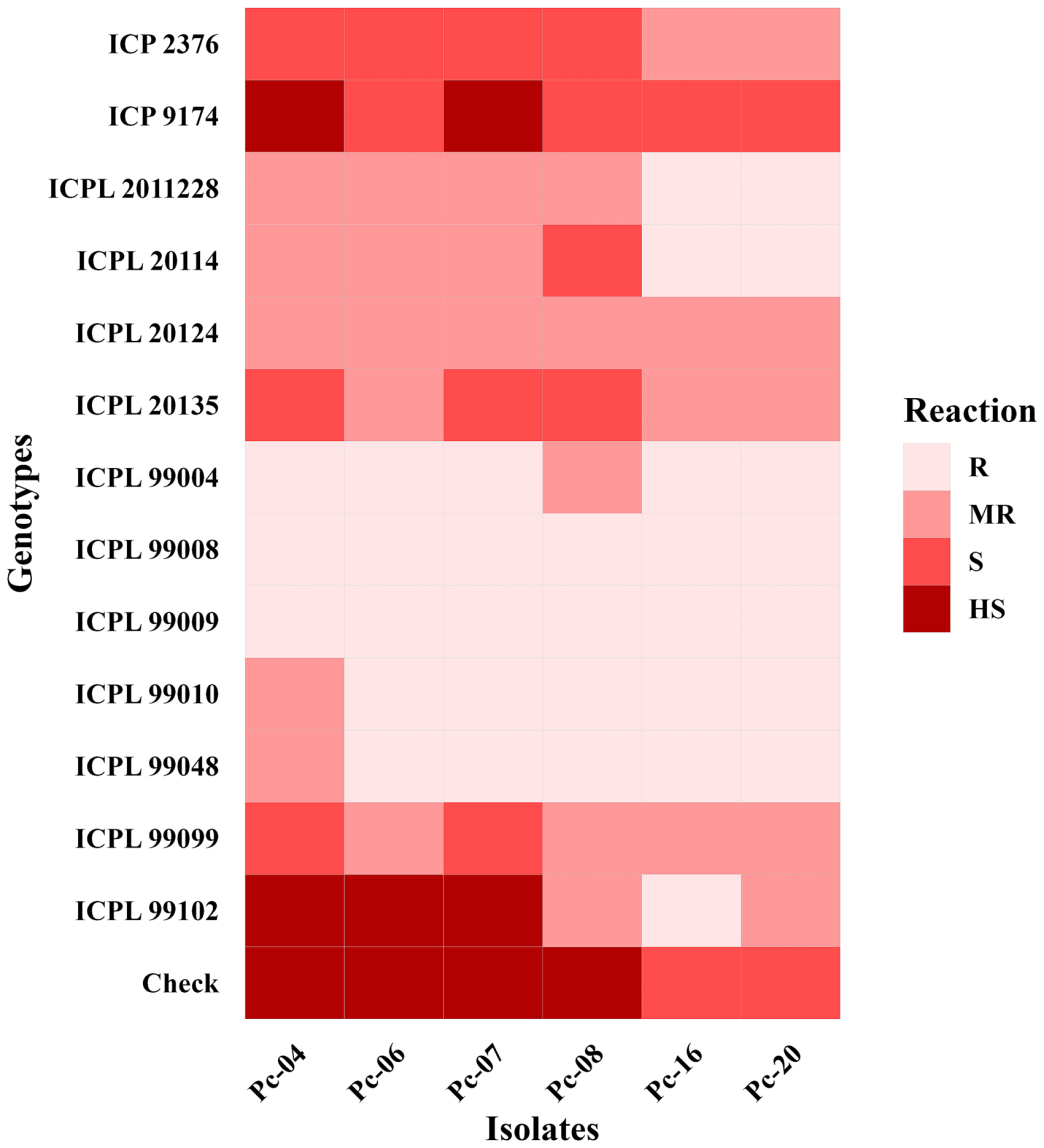
Heatmap visualization of reaction of *P. cajani* isolates across fourteen genotypes. The selected isolates of *P. cajani* were displayed by *x*-axes while the tested genotypes were displayed by the *y*-axes. The plot legend DI depicts the PDI rating in color.

**Figure 12:**
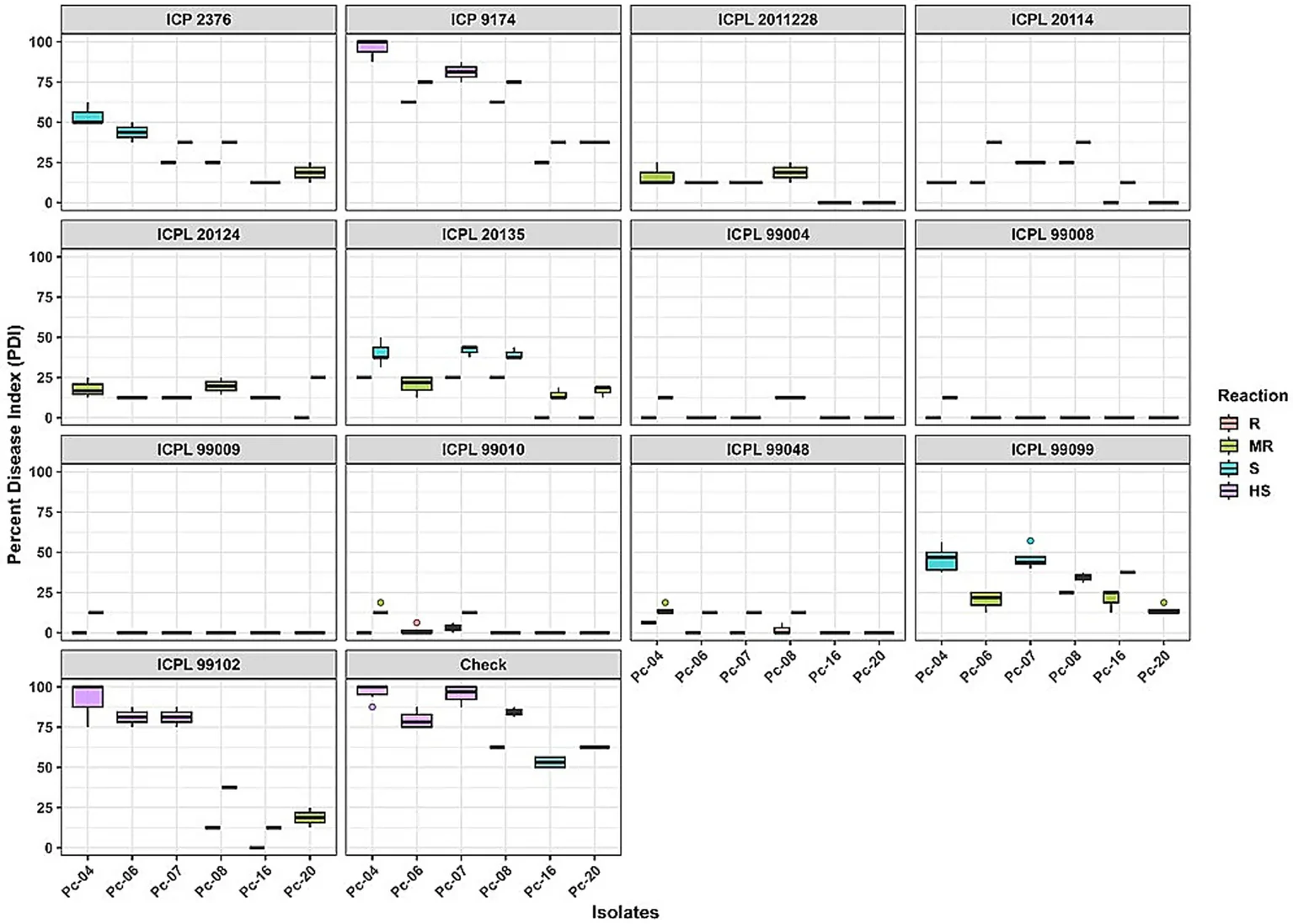
A box plot representation of pathogenic variability of six different isolates of *P. cajani* across fourteen diverse genotypes.

**Figure 13:**
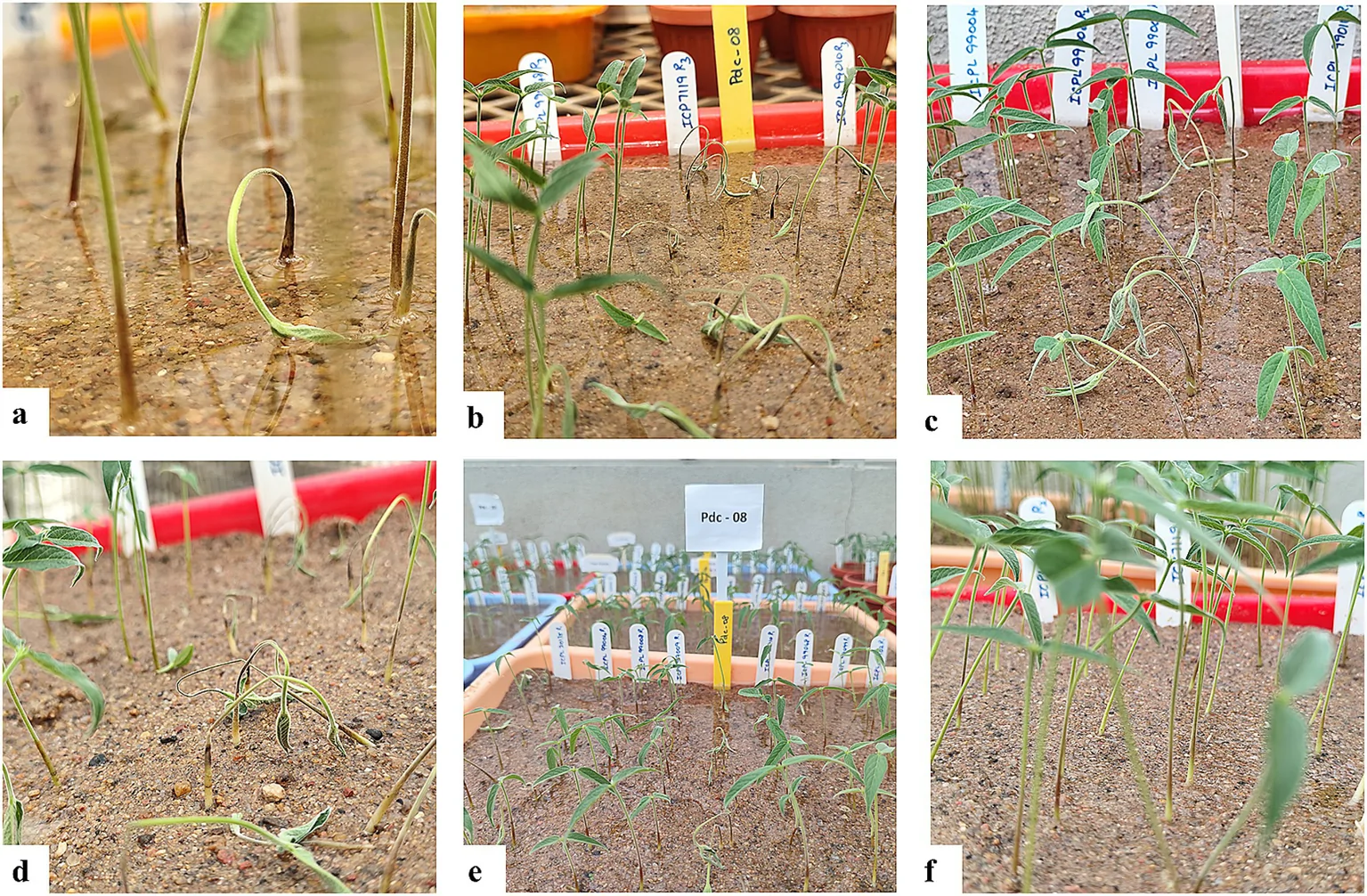
Symptom expression of diverse genotypes upon screening against *P. cajani* isolates. **(a–e)** Dark brown to black lesions on stem of susceptible genotypes and no/less infection on resistant genotypes; **(f)** Pathogen uninoculated control free of infection.

Several genotypes exhibited high resistance and low disease incidence across isolates (Figures 11, 12). ICPL 99008 and ICPL 99009 were found to be highly resistant (≤10% PDI) to all the isolates. ICPL 99004, ICPL 99010 and ICP 99048, despite returning overall mean PDI values consistent with a resistant classification (Table 7), exhibited moderately resistant reactions to one of the isolates in the tested panel and were therefore conservatively considered as moderately resistant in their final classification. The genotypes ICPL 2011228, ICPL 20114, ICPL 20124 and ICPL 20135, were also found to be moderately resistant (< 20% PDI). In contrast, ICP 9174 and ICPL 99102 were found susceptible to all the isolates, while ICP 2376 and ICPL 99099 exhibited isolate-specific susceptibility patterns. The susceptible check remained uniformly highly susceptible to all the isolates (Figure 13). Based on the pooled reactions, five genotypes were classified as Resistant (R), four as Moderately Resistant (MR), four as Susceptible (S), and one as Highly Susceptible (HS) (Table 6). But only two genotypes (ICPL 99008 and ICPL 99009), showed consistent resistant reactions against all the tested isolates. Collectively, these findings reveal strong host-pathogen specificity and identify multiple genotypes with broad-spectrum resistance that can serve as robust donor sources in PB resistance-breeding programs.

**Table 7:** Response of fourteen different pigeonpea genotypes against representative isolates of *P. cajani*.

S. No.	Genotypes	Pc-04	Pc-06	Pc-07	Pc-08	Pc-16	Pc-20	Mean
1	ICP 2376	S	S	S	S	MR	MR	S
2	ICP 9174	HS	S	HS	S	S	S	S
3	ICPL 2011228	MR	MR	MR	MR	R	R	MR
4	ICPL 20114	MR	MR	MR	S	R	R	MR
5	ICPL 20124	MR	MR	MR	MR	MR	MR	MR
6	ICPL 20135	S	MR	S	S	R	R	MR
7	ICPL 99004	R	R	R	MR	R	R	R
8	ICPL 99008	R	R	R	R	R	R	R
9	ICPL 99009	R	R	R	R	R	R	R
10	ICPL 99010	MR	R	R	R	R	R	R
11	ICPL 99048	MR	R	R	R	R	R	R
12	ICPL 99099	S	MR	S	MR	MR	MR	S
13	ICPL 99102	HS	HS	HS	MR	R	MR	S
14	ICP 7119 (Check)	HS	HS	HS	HS	S	S	HS

R-Resistant (0–10.0 PDI%); MR-Moderately Resistant (10.1–30.0 PDI %); S-Susceptible (30.1–70.0 PDI %) and HS-Highly Susceptible (70.1–100 PDI %).

## 4 Discussion

The present study provides an integrated assessment of morpho-cultural, physiological, molecular and pathogenic variability among *P. cajani* isolates and demonstrates considerable phenotypic and genetic heterogeneity within the pathogen population infecting pigeonpea in India. Understanding such variability is crucial because the durability of host resistance in pigeonpea is inherently linked to the evolutionary potential and ecological adaptability of the pathogen (McDonald and Linde, 2002; Karasov et al., 2020; Mohanapriya et al., 2025). Diversity within *P. cajani* populations has been proposed earlier (Sharma et al., 2015; Singh et al., 2017; Jadesha et al., 2022), but our work stands out among the few that associate variability and host-pathogen interactions to establish isolate-specific biological profiles, highlighting their implications for disease epidemiology and resistance breeding.

The isolation of *P. cajani* from pigeonpea fields across diverse agro-ecological zones of India accentuates the widespread occurrence and adaptive nature of the pathogen in varied soil environments. The recovery of 22 distinct isolates from red, black and sandy-loam soils indicates that *P. cajani* possesses considerable ecological flexibility, enabling it to persist and infect pigeonpea under different field conditions. Similar observations on wide geographical distribution and pathogen recovery across soil types have been reported for *P. cajani* infecting pigeonpea (Sharma et al., 2006; Pande et al., 2011), signifying broader ecological amplitude.

Marked differences in colony morphology, mycelial density, sporangial architecture and sporulation demonstrate considerable phenotypic disparity among the isolates. TEA, V8J and OMA supported the most vigorous growth, consistent with earlier studies (Singh et al., 2017; Jadesha et al., 2022), reporting that *Phytophthora* spp. establish on media rich in carbohydrates and organic acids. The inability of *P. cajani* to grow on RBA aligns with observations in other *Phytophthora* sp., where the antifungal components in rose bengal inhibit growth (Ruiz-Gómez and Miguel-Rojas, 2021; Abad et al., 2023). The robust growth of Pc-04 and Pc-07 across media suggests higher metabolic competence and substrate utilization efficiency, similar to the aggressive isolates reported for *P. capsici* (Lee et al., 2021). Conversely, the poor growth of Pc-08 on most media points to nutritional specialization or reduced competitive vigor (Trzewik et al., 2022). The predominance of non-papillate and non-caducous sporangia aligns with earlier taxonomic descriptions of *P. cajani* (Sharma and Ghosh, 2018; Jadesha et al., 2022). The observed range in sporangial dimensions (35.85–47.20 μm × 19.40–26.69 μm) and the presence of ovoid, obovoid, obpyriform to elongated forms are consistent with phenotypic modulation reported for other soilborne *Phytophthora* spp. (Erwin and Riberiro, 1996; Abad et al., 2023). Sporulation vigor was substantially isolate-dependent, rapid sporulating isolates (Pc-04, Pc-07) are typically associated with higher epidemic potential, as high sporangial production magnifies inoculum load (Widmer, 2015).

The pH and temperature tolerance of isolates indicated clear physiological variability, reflecting their capacity to survive across diverse soil environments. All isolates exhibited poor growth under strongly acidic conditions, with complete inhibition at pH 3.5 and severely reduced growth at pH 4.0, indicating that pronounced acidic environment disrupts key enzymatic and membrane functions (Liu et al., 2018). Growth increased steadily between pH 4.5 and 5.5, marking a physiological threshold commonly observed in oomycetes, where nutrient uptake become favorable. Most isolates achieved optimal growth between pH 5.5 and 8.0, consistent with the slightly acidic to near-neutral range typically favorable for *Phytophthora* spp., while the modest decline beyond pH 8.0 reflects reduced suitability of alkaline conditions, in agreement with earlier reports (Kong et al., 2009; Dasgupta et al., 2012). To our knowledge, this is the first study to concurrently characterize the pH responses of *P. cajani* in terms of both sporulation behavior and mycelial growth. Temperature responses further showed that *P. cajani* is confined to warm, humid environments, with optimal growth at 25–30 °C in agreement with earlier reports (Mishra et al., 2010). However, unlike the results of Jadesha et al. (2022) which documented nearly 40 mm radial growth at 15 °C, our isolates showed no growth at this temperature, with mycelial development initiating only at 17 °C. The reversible growth suppression observed after low-temperature exposure indicates a dormant survival mechanism, enabling the pathogen to persist until favorable temperatures return. The reversible suppression of growth at 5–15 °C contrasts with the irreversible lethality at ≥38 °C, indicating that *P. cajani* behaves similarly to the related species like *P. sojae, P. polonica* and *P. hydropathica* (Tyler, 2007; Abad et al., 2023; Christova, 2024). The lethal effect of 40 °C spotlights potential natural temperature limits, although microclimatic soil cooling during monsoon flooding likely protects the pathogen during heatwaves. Notably, Pc-04 and Pc-07 showed the reliable physiological responsiveness that might contribute to their higher aggressiveness. Such information holds meaningful relevance for understanding the ecology of the pathogen and optimizing *in vitro* culture conditions for further physiological and pathogenicity studies.

The successful amplification and sequencing of the ITS-5.8S rDNA region, subsequently confirmation via BLASTn analysis, explicitly established the identity of all isolates as *P. cajani*. It is evident from the phylogenetic tree that all *P. cajani* isolates formed a single, well-defined cluster, strongly supported by a bootstrap value of 100%, confirming the genetic cohesiveness of the species. ITS based phylogeny clustered all the *P. cajani* isolates intact and remarked the distinct separation of *P. cajani* isolates from distant taxa such as *P. infestans* and *P. capsici*, in accordance with the previous phylogenetic establishments (Yang et al., 2017; Coomber et al., 2023). All the Phytophthora isolates clustered in respective clades as per the taxonomy given by Abad et al. (2023). While the ITS region proved effective for species confirmation, the limited intraspecific variation observed suggests that additional loci may be required to resolve finer-scale genetic diversity within *P. cajani* populations.

The pathogenicity evaluation of fourteen pigeonpea genotypes against diverse isolates of *P. cajani* revealed a broad spectrum of host responses, ranging from highly susceptible to highly resistant. Isolates Pc-04 and Pc-07 consistently exhibited high PDI values across multiple genotypes, reflecting their aggressive nature and correlating with their superior mycelial growth, prolific sporulation and pronounced physiological resilience. In contrast, Pc-16 and Pc-20 exhibited moderate aggressiveness, highlighting intraspecific differences in pathogenic potential. These patterns reinforce the dynamic nature of *P. cajani* populations and the need for continuous monitoring of aggressive patterns and are in line with studies related to *P. sojae* by Cerritos-Garcia et al. (2023). The analysis of isolate × genotype interactions further disclosed that disease expression in pigeonpea is largely determined by specific host-pathogen. Notably, ICPL 99008 and ICPL 99009 exhibited consistently low PDI values (≤10%) irrespective of the inoculated isolate, identifying them as strong and broad spectrum resistance sources. Conversely, certain genotypes showed isolate-dependent reactions, such as ICPL 99004, ICPL 99010 and ICPL 99048 being moderately susceptible to either Pc-08 or Pc-04, indicate possible pathotype existence, similar to patterns reported in *P. sojae and P. medicaginis* (Bithell et al., 2022; Heo et al., 2024). Moderate but, reliable resistance observed in ICPL 2011228, ICPL 20114, ICPL 20124 and ICPL 20135 indicates partial resistance mechanisms. These mechanisms might entail reduced infection efficiency, slower disease progression or delayed colonization, providing a valuable buffer against aggressive isolates. The uniform susceptibility of ICP 7119 validates its inclusion as a reliable susceptible check and unequivocally reinforces its suitability for screening programs. Given that resistance to PB is often isolate-dependent and environmentally unstable (Pande et al., 2011; Sharma and Ghosh, 2018), the stable resistance profiles identified in this study are particularly noteworthy. These genotypes represent valuable genetic resources for pigeonpea breeding programs aiming to develop cultivars with broad-spectrum and durable resistance, thereby contributing to more sustainable disease management strategies and long-term crop productivity.

In conclusion, our study provides one of the most detailed and integrative assessments of *P. cajani* biology to date. The pronounced variability across morpho-cultural, physiological and pathogenic traits demonstrates a highly dynamic pathogen population capable of adapting to diverse environments. Coupled with clear host genotype-specific interactions, these findings highlight the importance of deploying diverse and durable resistance sources. The consistently high resistance observed in ICPL 99008 and ICPL 99009 highlights their potential for use as donor parents in future pigeonpea breeding programs to improve resistance to *P. cajani*. Further evaluation under diverse environments and against a broader range of pathogen isolates will help confirm their wider utility. The multi-dimensional variability uncovered in this study provides a foundational framework for epidemiological modeling, climate-responsive disease forecasting, and the development of environmentally tailored PB management strategies. Future integration of transcriptomic profiling, effector characterization and resistance-gene mapping will be pivotal to unravel the molecular basis of host–pathogen compatibility and for designing next-generation pigeonpea cultivars resilient to evolving *Phytophthora* populations.

## Data Availability

The datasets presented in this study can be found in online repositories. The names of the repository/repositories and accession number(s) can be found in the article/Supplementary material.
