# Supplementary material for: Pigeonpea—Phytophthora cajani isolates characterization and assessment of resistance in elite pigeonpea germplasm and breeding lines
Source: Front Microbiol. 2026 Aug 12;17:1910854. doi: 10.3389/fmicb.2026.1910854 (PMC13506769; doi:10.3389/fmicb.2026.1910854)
Supplement: Supplementary file 1 [file Data_Sheet_1.docx]

**Supplementary Table 1. Cultural variability of six selected *P. cajani* isolates on different solid media.**

| **Isolates** | **Mycelial growth (mm) in different solid media** | | | | | | | | | |
| --- | --- | --- | --- | --- | --- | --- | --- | --- | --- | --- |
|  | **CDX** | **CMA** | **MG** | **OMA** | **PAM** | **PDA** | **RBA** | **TEA** | **V8J** | **Mean** |
| **Pc - 04** | 27.67 ± 2.84 (5.26) | 69.33 ± 2.47 (8.33) | 23.50 ± 1.32 (4.85) | 80.17 ± 0.58 (8.95) | 33.50 ± 0.50 (5.79) | 67.00 ± 3.04 (8.19) | _ | 90.00 ± 0 (9.49) | 82.67 ± 1.89 (9.09) | **59.23^a^** |
| **Pc - 06** | 14.33 ± 2.31 (3.79) | 32.83 ± 3.06 (5.73) | 15.17 ± 0.29 (3.89) | 51.83 ± 0.76 (7.2) | 18.50 ± 0.50 (4.3) | 21.67 ± 1.61 (4.65) | _ | 82.83 ± 2.08 (9.1) | 63.17 ± 5.30 (7.95) | **37.54^d^** |
| **Pc - 07** | 37.50 ± 2.65 (6.12) | 70.33 ± 2.84 (8.39) | 21.83 ± 0.76 (4.67) | 80.33 ± 1.26 (8.96) | 38.83 ± 0.76 (6.23) | 58.33 ± 0.29 (7.64) | _ | 90.00 ± 0 (9.49) | 82.00 ± 0.87 (9.06) | **59.90^a^** |
| **Pc - 08** | 31.33 ± 2.57 (5.6) | 49.83 ± 3.21 (7.06) | 15.83 ± 0.29 (3.98) | 42.83 ± 0.76 (6.54) | 45.5 0± 0.50 (6.75) | 30.00± 2.18 (5.48) | _ | 65.83 ± 1.04 (8.11) | 48.00 ± 1.50 (6.93) | **41.15^cd^** |
| **Pc - 16** | 23.17 ± 4.51 (4.81) | 55.83 ± 2.89 (7.47) | 30.67 ± 0.58 (5.54) | 63.00 ± 1.32 (7.94) | 43.50 ± 0.50 (6.6) | 52.33 ± 2.47 (7.23) | _ | 71.17± 2.08 (8.44) | 57.50 ± 2.00 (7.58) | **49.65^b^** |
| **Pc - 20** | 28.00 ± 3.46 (5.29) | 42.23 ± 1.17 (6.5) | 15.50 ± 0.50 (3.94) | 58.77 ± 1.12 (7.67) | 46.70 ± 1.08 (6.83) | 32.00 ± 2.78 (5.66) | _ | 73.83 ± 1.26 (8.59) | 56.33 ± 2.25 (7.51) | **44.17^c^** |
| **Mean** | **27.00^f^** | **53.40^c^** | **20.42^f^** | **62.82^b^** | **37.76^e^** | **43.56^d^** | **_** | **78.94^a^** | **64.94^b^** | _ |

Data represent mean ± standard deviation. Figures in the parenthesis are square root transformed values. “_" represent no growth. Means followed by common letters are not significantly different at p ≤ 0.05 by Duncan’s multiple range test (DMRT). CDX - Czapek dox agar; CMA -Corn Meal Agar; **MGA – Malachite Green Agar; OMA – Oatmeal Agar; PA – Peptone Agar; PDA – Potato Dextrose Agar; RBA – Rose Bengal Agar; TEA – Tomato Extract Agar; V8J – Vegetable 8 Juice Agar**

**Supplementary Table 2. Morphological characterisation of *P. cajani* isolates.**

| **S.No.** | **Isolate Name** | **Rate of Sporulation** | **Length of sporangia (µm)** | **Width of sporangia**  **(µm)** | **Sporangial circumference**  **(µm)** | **Hyphal swellings** | **Chlamydospores** | **Abundance of zoospores** |
| --- | --- | --- | --- | --- | --- | --- | --- | --- |
| 1 | Pc-04 | *** | 47.20 | 26.69 | 142.44 | + | **−** | A |
| 2 | Pc-06 | ** | 38.31 | 23.11 | 116.98 | + | **−** | A |
| 3 | Pc-07 | *** | 39.16 | 23.26 | 131.09 | + | **−** | A |
| 4 | Pc-08 | ** | 35.85 | 19.87 | 114.75 | + | **−** | A |
| 5 | Pc-16 | * | 36.70 | 19.40 | 109.81 | + | **−** | S |
| 6 | Pc-20 | * | 36.90 | 22.38 | 113.70 | + | **−** | S |

Rate of sporulation- Very Fast (*******), Fast (******), Moderate (*****); Hyphal swellings / Chlamydospores- Absent (**−**), Present (**+**); Abundance of zoospores- Abundant (A), Sparse (S).

**Supplementary Table 3. Effect of different media pH on diverse isolates of *P. cajani***

| **Isolates** | **Mycelial growth (mm) in different media pH** | | | | | | | | | | | | | | | |
| --- | --- | --- | --- | --- | --- | --- | --- | --- | --- | --- | --- | --- | --- | --- | --- | --- |
|  | **3.5** | **4** | **4.5** | **5** | **5.5** | **6** | **6.5** | **7** | **7.5** | **8** | **8.5** | **9** | **9.5** | **10** | **10.5** | **Mean** |
| **Pc - 04** | _ | 11.67 ± 0.58 (0.35) | 72.50 ± 0.50 (1.02) | 80.77 ± 0.25 (1.12) | 90.00 ± 0.00 (1.25) | 90.00 ± 0.00 (1.25) | 90.00 ± 0.00 (1.25) | 90.00 ± 0.00 (1.25) | 90.00 ± 0.00 (1.25) | 90.00 ± 0.00 (1.25) | 75.17 ± 2.47 (1.05) | 67.00 ± 1.50 (0.96) | 48.50 ± 0.87 (0.77) | 44.33 ± 0.76 (0.73) | 42.17 ± 2.08 (0.71) | **70.15^a^** |
| **Pc - 06** | _ | 27.50 ± 5.63 (0.55) | 58.17 ± 1.53 (0.87) | 58.67 ± 1.61 (0.87) | 79.83 ± 0.76 (1.11) | 81.17 ± 0.76 (1.12) | 80.33 ± 0.29 (1.11) | 82.50 ± 0.00 (1.14) | 79.43 ± 0.40 (1.10) | 76.17 ± 0.29 (1.06) | 52.33 ± 1.04 (0.81) | 42.50 ± 1.00 (0.71) | 39.00 ± 1.32 (0.67) | 35.33 ± 2.93 (0.64) | 34.33 ± 2.57 (0.63) | **59.09^b^** |
| **Pc - 07** | _ | 11.47 ± 0.06 (0.35) | 68.83 ± 1.53 (0.98) | 80.00 ± 0.50 (1.11) | 90.00 ± 0.00 (1.25) | 90.00 ± 0.00 (1.25) | 90.00 ± 0.00 (1.25) | 90.00 ± 0.00 (1.25) | 90.00 ± 0.00 (1.25) | 90.00 ± 0.00 (1.25) | 72.83 ± 2.89 (1.02) | 67.00 ± 1.50 (0.96) | 47.27 ± 2.05 (0.76) | 43.33 ± 0.76 (0.72) | 40.83 ± 1.04 (0.69) | **69.40^a^** |
| **Pc - 08** | _ | 18.80 ± 0.26 (0.45) | 44.50 ± 0.50 (0.73) | 48.57 ± 0.60 (0.77) | 60.33 ± 0.76 (0.89) | 59.83 ± 2.57 (0.88) | 60.17 ± 1.76 (0.89) | 61.33 ± 2.08 (0.90) | 59.17 ± 0.76 (0.88) | 58.83 ± 0.76 (0.87) | 52.50 ± 2.18 (0.81) | 41.60 ± 0.85 (0.70) | 40.97 ± 1.45 (0.69) | 19.62 ± 0.65 (0.46) | 18.50 ± 0.50 (0.44) | **46.05^c^** |
| **Pc - 16** | _ | 23.33 ± 2.57 (0.50) | 43.33 ± 0.29 (0.72) | 63.57 ± 1.44 (0.92) | 73.73 ± 0.25 (1.03) | 76.17 ± 0.76 (1.06) | 74.00 ± 0.50 (1.04) | 74.67 ± 1.26 (1.04) | 75.10 ± 0.85 (1.05) | 75.00 ± 1.32 (1.05) | 69.57 ± 1.01 (0.99) | 51.83 ± 1.26 (0.80) | 50.17 ± 0.76 (0.79) | 34.17 ± 1.04 (0.62) | 33.83 ± 2.57 (0.62) | **58.46^b^** |
| **Pc - 20** | _ | 16.33 ± 2.57 (0.42) | 44.17 ± 0.76 (0.73) | 56.00 ± 2.00 (0.85) | 75.00 ± 0.50 (1.05) | 75.67 ± 1.04 (1.05) | 74.33 ± 0.76 (1.04) | 74.17 ± 1.04 (1.04) | 76.83 ± 1.26 (1.07) | 75.33 ± 1.04 (1.05) | 61.67 ± 1.04 (0.90) | 43.50 ± 1.00 (0.72) | 41.67 ± 1.61 (0.70) | 41.17 ± 1.53 (0.70) | 40.00 ± 1.32 (0.68) | **56.85^bc^** |
| **Mean** | _ | **18.18^g^** | **55.25^c^** | **64.59^b^** | **78.15^a^** | **78.81^a^** | **78.14^a^** | **78.78^a^** | **78.42^a^** | **77.56^a^** | **64.01^b^** | **52.24^cd^** | **44.59^de^** | **36.33^ef^** | **34.94^f^** | **_** |

Data represent mean ± standard deviation. Figures in the parenthesis are square root transformed values. “--" represent no growth. Means followed by common letters are not significantly different at p ≤ 0.05 by Duncan’s multiple range test (DMRT).

**Supplementary Table 4. Effect of different incubation temperature on diverse isolates of *P. cajani***

| **Isolates** | **Mycelial growth (mm) under different temperature** | | | | | | | | |
| --- | --- | --- | --- | --- | --- | --- | --- | --- | --- |
|  | **5** | **10** | **15** | **20** | **25** | **30** | **35** | **40** | **Mean** |
| **Pc - 04** | _ | _ | _ | 53.67 ± 1.04  (7.33) | 90.00 ± 0.00  (9.49) | 90.00 ± 0.00  (9.49) | 50.27 ± 0.75  (7.09) | _ | **70.98^a^** |
| **Pc - 06** | _ | _ | _ | 36.67 ± 5.20  (6.06) | 83.83 ± 2.57  (9.16) | 83.50 ± 0.50  (9.14) | 51.50 ± 6.08  (7.18) | _ | **63.88^bc^** |
| **Pc - 07** | _ | _ | _ | 52.17 ± 0.76  (7.22) | 90.00 ± 0.00  (9.49) | 84.33 ± 0.76  (9.18) | 43.53 ± 0.72  (6.6) | _ | **67.51^ab^** |
| **Pc - 08** | _ | _ | _ | 24.17 ± 4.25  (4.92) | 61.00 ± 3.50  (7.81) | 66.50 ± 4.77  (8.15) | 49.50 ± 3.28  (7.04) | _ | **50.29^d^** |
| **Pc - 16** | _ | _ | _ | 29.83 ± 1.04  (5.46) | 77.33 ± 2.47  (8.79) | 79.03 ± 1.79  (8.89) | 48.00 ± 3.91  (6.93) | _ | **58.55^c^** |
| **Pc - 20** | _ | _ | _ | 28.50 ± 2.00  (5.34) | 75.17 ± 0.29  (8.67) | 79.33 ± 1.89  (8.91) | 32.83 ± 2.36  (5.73) | _ | **53.96^d^** |
| **Mean** | _ | _ | _ | **37.50^c^** | **79.56^a^** | **80.45^a^** | **45.94^b^** | _ | **_** |

Data represent mean ± standard deviation. Figures in the parenthesis are square root transformed values. “--" represent no growth. Means followed by common letters are not significantly different at p ≤ 0.05 by Duncan’s multiple range test (DMRT).

**Supplementary Table 5. PB Disease reaction of fourteen different pigeonpea genotypes against representative isolates of *P. cajani.***

| **Isolates**  **Genotypes** | **Percent Disease Incidence-PDI (%)** | | | | | | |
| --- | --- | --- | --- | --- | --- | --- | --- |
|  | **Pc-04** | **Pc-06** | **Pc-07** | **Pc-08** | **Pc-16** | **Pc-20** | **Mean** |
| **ICP 2376** | 54.17 ± 7.22  (0.83) | 43.75 ± 8.84 (0.72) | 31.25 ± 8.84  (0.59) | 31.25 ± 8.84  (0.59) | 12.5 ± 0  (0.36) | 18.75 ± 8.84  (0.44) | 33.65 |
| **ICP 9174** | 95.83 ± 7.22  (1.45) | 68.75 ± 8.84 (0.98) | 81.25 ± 8.84  (1.13) | 68.75 ± 8.84  (0.98) | 31.25 ± 8.84  (0.59) | 37.5 ± 0  (0.66) | 66.35 |
| **ICPL 2011228** | 16.67 ± 7.22  (0.42) | 12.5 ± 0 (0.36) | 12.5 ± 0  (0.36) | 18.75 ± 8.84  (0.44) | 0 ± 0 (0) | 0 ± 0 (0) | 10.58 |
| **ICPL 20114** | 12.5 ± 0  (0.36) | 25 ± 17.68 (0.51) | 25 ± 0  (0.52) | 31.25 ± 8.84  (0.59) | 6.25 ± 8.84  (0.18) | 0 ± 0 (0) | 16.35 |
| **ICPL 20124** | 18.06 ± 6.36  (0.44) | 12.5 ± 0 (0.36) | 12.5 ± 0  (0.36) | 19.64 ± 7.58  (0.46) | 12.5 ± 0  (0.36) | 12.5 ± 17.68  (0.26) | 14.88 |
| **ICPL 20135** | 37.5 ± 8.84  (0.66) | 20.31 ± 5.98 (0.46) | 37.5 ± 8.84  (0.66) | 35.94 ± 7.86  (0.64) | 10.94 ± 7.86  (0.29) | 12.5 ± 8.84  (0.31) | 26.68 |
| **ICPL 99004** | 4.17 ± 7.22  (0.12) | 0 ± 0 (0) | 0 ± 0 (0) | 12.5 ± 0  (0.36) | 0 ± 0 (0) | 0 ± 0 (0) | 2.88 |
| **ICPL 99008** | 4.17 ± 7.22  (0.12) | 0 ± 0 (0) | 0 ± 0 (0) | 0 ± 0 (0) | 0 ± 0 (0) | 0 ± 0 (0) | 0.96 |
| **ICPL 99009** | 4.17 ± 7.22  (0.12) | 0 ± 0 (0) | 0 ± 0 (0) | 0 ± 0 (0) | 0 ± 0 (0) | 0 ± 0 (0) | 0.96 |
| **ICPL 99010** | 11.46 ± 6.14  (0.32) | 1.56 ± 3.12 (0.06) | 7.81 ± 5.98  (0.24) | 0 ± 0 (0) | 0 ± 0 (0) | 0 ± 0 (0) | 4.09 |
| **ICPL 99048** | 11.46 ± 4.7  (0.34) | 3.12 ± 6.25 (0.09) | 9.38 ± 6.25  (0.27) | 4.69 ± 5.98  (0.15) | 0 ± 0 (0) | 0 ± 0 (0) | 5.29 |
| **ICPL 99099** | 45.83 ± 7.57  (0.74) | 20.31 ± 5.98 (0.46) | 46.16 ± 7.53  (0.75) | 29.69 ± 5.98  (0.57) | 25 ± 10.21  (0.52) | 14.06 ± 3.12  (0.38) | 31.38 |
| **ICPL 99102** | 91.67 ± 14.43  (1.4) | 81.25 ± 8.84 (1.13) | 81.25 ± 8.84  (1.13) | 25 ± 17.68  (0.51) | 6.25 ± 8.84  (0.18) | 18.75 ± 8.84  (0.44) | 53.85 |
| **ICP 7119 (Check)** | 96.88 ± 5.23  (1.47) | 79.69 ± 5.98 (1.11) | 95.31 ± 5.98  (1.42) | 73.44 ± 12.88  (1.04) | 53.12 ± 3.61  (0.82) | 62.5 ± 0  (0.91) | 78.37 |
| **Mean** | 37.24 | 25.99 | 33.48 | 26.03 | 12.99 | 13.98 | _ |

Data represent mean ± standard deviation. Figures in the parenthesis are arc sine transformed values.
